# LINE-1-like retrotransposons contribute to RNA-based gene duplication in dicots

**DOI:** 10.1038/srep24755

**Published:** 2016-04-21

**Authors:** Zhenglin Zhu, Shengjun Tan, Yaqiong Zhang, Yong E. Zhang

**Affiliations:** 1 School of Life Sciences, Chongqing University, Chongqing 400044, China; 2 Key Laboratory of the Zoological Systematics and Evolution & State Key Laboratory of Integrated Management of Pest Insects and Rodents, Institute of Zoology, Chinese Academy of Sciences, Beijing 100101, China; 3 University of Chinese Academy of Sciences, Beijing 100049, China

## Abstract

RNA-based duplicated genes or functional retrocopies (retrogenes) are known to drive phenotypic evolution. Retrogenes emerge via retroposition, which is mainly mediated by long interspersed nuclear element 1 (LINE-1 or L1) retrotransposons in mammals. By contrast, long terminal repeat (LTR) retrotransposons appear to be the major player in plants, although an L1-like mechanism has also been hypothesized to be involved in retroposition. We tested this hypothesis by searching for young retrocopies, as these still retain the sequence features associated with the underlying retroposition mechanism. Specifically, we identified polymorphic retrocopies (retroCNVs) by analyzing public Arabidopsis (*Arabidopsis thaliana*) resequencing data. Furthermore, we searched for recently originated retrocopies encoded by the reference genome of Arabidopsis and *Manihot esculenta*. Across these two datasets, we found cases with L1-like hallmarks, namely, the expected target site sequence, a polyA tail and target site duplications. Such data suggest that an L1-like mechanism could operate in plants, especially dicots.

Rapidly accumulating evidence demonstrates that new genes play diverse functional roles and serve as a major driver of phenotypic evolution^1^. One important mechanism to create these lineage- or species-specific genes is RNA-based duplication or retroposition^1^, in which an mRNA template is reverse transcribed by retrotransposons and subsequently reinserted into the genome as a functional retrocopy or retrogene^2^. The identification of retrogenes is straightforward given the hallmark of intron loss relative to the parental copies. Moreover, because of the loss of most of the preexisting regulatory sequences, retrogenes are predicted to be subject to neofunctionalization, *i.e*., to play a different function compared to their parental genes^3^. Thus, retrogenes have been an attractive research target for decades. For example, one of the first reported new genes, *jingwei* in *Drosophila*, is a retrogene^4^. In plants, genome-wide surveys performed by others and ourselves have identified numerous retrogenes in Arabidopsis (*Arabidopsis thaliana*), rice, and so on^5^,^6^,^7^. Although the majority of these retrogenes are functionally uncharacterized, *Sun* is known to underlie morphological variation of the tomato fruit^8^, while *CYP98A8* and *CYP98A9* are involved in pollen development in Arabidopsis^9^.

Retrotransposons provide the enzymatic machinery for retroposition and can be divided into various orders, including long interspersed nuclear elements (LINEs) and long terminal repeat (LTR) retrotransposons, among others^10^. LINEs are more abundant in animals, whereas LTR retrotransposons are dominant in plants^10^. LINE-1 or L1 appears to be the exclusive driver of retroposition in mammals^2^,^11^. A typical L1-mediated insertion is accompanied with sequence features such as a target site (TTAAAA), target site duplication (TSD) and a 3′ polyA tail^2^,^12^,^13^. Notably, a nick is created between “TT” and “AAAA” during the retroposition, and the later was further used to prime the reverse transcription of mRNA. So, “AAAA” is also duplicated in this aspect. As a conventional terminology, “AAAA” is generally treated as a part of the polyA tail rather than TSD^2^. However, these three features are not always all present. For example, L1-mediated tailless retrocopies are found in therian genomes, where the polyA tail is absent but the target site and TSD are still present^14^. In contrast, in plants, retrogenes are mostly flanked by LTR retrotransposons, including the aforementioned *Sun* locus in tomato^8^, *Bs1* in maize^15^,^16^ and a dozen retrogenes in rice^5^, suggesting that retroposition in these species is mainly mediated by LTR retrotransposons. Interestingly, although not as abundant as in mammals, plant genomes are known to encode L1-clade LINEs^17^, and thus, L1 retrotransposons are hypothesized to drive retroposition in plants due to the relaxed recognition of template RNAs^18^. In the present study, we provide evidence that L1-like retrotransposons mediate the creation of retrocopies in plants, especially dicots.

## Results

To investigate the mechanism of retroposition in plants, we focused on the model species, Arabidopsis, due to the following two reasons: 1) the reference genome encodes up to 251 retrogenes^7^; 2) although Arabidopsis consists of more LTR retrotransposons than LINEs^19^, no retrogenes were reported to be flanked by LTR retrotransposons^20^, suggesting that LINE-mediated retroposition could occur. Because the sequence features associated with retroposition (*e.g.*, polyA tail or flanking LTRs) rapidly degenerate due to the accumulation of secondary mutations^2^, we focused on young retrocopies. Specifically, we took advantage of the next-generation sequencing data for 18 Arabidopsis accessions^21^ and searched for polymorphic retrocopies or retro-copy-number-variants (retroCNVs), followed by targeted local *de novo* assembly (see the Materials and Methods section). We were able to assemble the full-length sequences and corresponding flanking regions of four retroCNVs (Table 1), whose population frequencies ranged from 1/18 to 14/18 (Supplementary Tables S1 and S2 and Fig. S1A–D). On the basis of the assembled sequences, we validated all four retroCNVs and flanking sequences with PCR (Supplementary Fig. S1E,F). These retroCNVs are located on different chromosomes from their parental genes (Table 1), which is consistent with the between-chromosome duplication preference of retroposition^22^ but stands in contrast to the within-chromosome bias of DNA-level duplication^23^.

**Figure 1:**
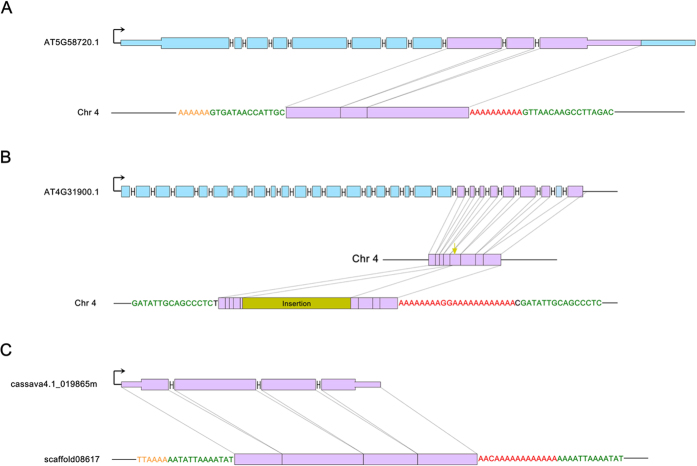
Schematic representation of three retrocopies. Thick and thin boxes stand for the coding region and the untranslated region, respectively. The symbol like “H” refers to intron. The exon size is roughly drawn to scale. The arrow means the transcription direction. The dashed line shows the correspondance of sequences between the parental gene and the retroCNV. The retroposed segment is marked in purple with the other in light blue. For the retroCNV, the candidate target site, target site duplication and polyA tail are marked in orange, green, and red, respectively. Panel (**A**) shows the retroCNV derived from the parental gene AT5G58720.1 in Arabidopsis where a partial sequence derived from the last three exons of AT5G58720.1 was retroposed and inserted into Chromosome 4 (Chr 4). Panel (**B**) shows a retrocopy encoded by the Arabidopsis reference genome, and Panel (**C**) shows a retrocopy encoded by the *M. esculenta* reference genome. Interestingly, in Panel (**B**), an insertion of LTR retrotransposon (*Copia*) occurrs in the middle of the retrocopy, which is marked in dark yellow.

We then examined the sequence features associated with the four retroCNVs to identify the underlying retroposition mechanism. We found one retroCNV (RC_AT3G08580.2, retroCNV derived from AT3G08580.2) flanked by two *ATRAN*-type LTR retrotransposon segments, suggesting an LTR retrotransposon-mediated mechanism (Supplementary Fig. S2). Interestingly, this retroCNV encodes one intron inherited from its parental gene (Supplementary Fig. S2C), which is consistent with pervasive intron retention events in plants due to alternative splicing^24^,^25^. In contrast, no LTR retrotransposons were associated with the other three retroCNVs. Specifically, RC_AT5G58720.1 has features similar to those of retrogenes created by L1s (Fig. 1A and Supplementary Fig. S3): a candidate 10-bp polyA tail, a potential 17-bp TSD (identity of 61%, BLAST *E* = 0.45) and a possible target site “AAAAAA”, which is similar to the canonical “TTAAAA” motif. These data are consistent with the swift degeneration of flanking sequences after retroposition^2^,^26^. Since its putative TSD is too variable, we inferred that this site is possibly not a real TSD but rather encoded by the reference genome or accessions not encoding the retroCNV. In order to test this possibility, we downloaded all the assembled sequences of the 18 accessions, and investigated the insertion site in the accessions with RC_AT5G58720.1 genotyped as absent. We only found a single TSD (3′ TSD) rather than 5′ TSD and polyA at the insert site across the reference genome and 15 accessions with this retroCNV genotyped as absent (Supplementary Fig. S4A). Such a result suggests that RC_AT5G58720.1 is mediated by the L1-like mechanism.

RC_AT3G06040.1 has analogous features as RC_AT5G58720.1, but the polyA tail is short (4 bp) with an A-to-T substitution and a 33-bp TSD shows only 46% identity (*E* = 0.15, Supplementary Fig. S5). More than that, different from RC_AT5G58720.1, both the TSDs and polyA tail can be found across the reference genome and all accessions with this retroCNV genotyped as absent (Supplementary Fig. S4B). Thus, the inference of underlying mechanism is impossible. Interestingly, in all the accessions without RC_AT3G06040.1, there is an insertion around 6 kb between the two “TSDs”, which is replaced by RC_AT3G06040.1 in Can-0. Since the orthologous region in the closely related species, *Arabidopsis lyrata*, does not encode either the insertion or the retroCNV (Supplementary Fig. S6), whether the insertion predates the retroCNV or vice versa is unknown.

Finally, the fourth retroCNV, RC_AT5G51410.2, is peculiar in that the retroposed sequence is inserted into the reference genome without any L1-like or LTR features (Supplementary Fig. S7). In other words, no polyA tail, TSD, target motif or LTR retrotransposon remnant exists, suggesting either an additional, previously unknown mechanism or the rapid degeneration of the aforementioned hallmark sequences.

If Arabidopsis retroCNVs show hallmarks typical of L1-mediated retrogenes, a second type of young retrocopies, *i.e*., recently evolved retrocopies encoded by the reference genome, may also possibly harbor these features. By improving on the previous work^6^,^7^,^20^, we identified 10 retrocopies with an overall identity higher than 95% relative to the parental loci (Materials and Methods) including six entries covered in^6^,^7^,^20^ and four novel ones (Supplementary Table S3). Because all of these retrocopies are shared by at least 17 of the 18 Arabidopsis populations (Supplementary Table S4), they are very likely older than the retroCNVs, and thus the hallmarks associated with L1s may have already disappeared. Of the 10 retrocopies, we were able to identify the retroposition mechanism for four of them: one is associated with LTR retrotransposons, and the other three exhibit L1-like hallmarks (Supplementary Table S3). Specifically, R_AT4G31900.1 harbors a 22-bp polyA with only two A-to-G substitutions and a 15-bp TSD with high identity (93%, *E* = 3 × 10^−5^, only one mismatch). Interestingly, a 1.8-kb insertion (LTR element *Copia-82_ALY-I*) is situated in the middle (Fig. 1B and Supplementary Fig. S8), suggesting a secondary mutation after the L1-mediated retroposition. Similarly, R_AT1G05890.1 and R_AT4G21660.1 encode a 6-bp and 15-bp polyA tail, respectively, although no TSD was observed (Supplementary Figs S9 and S10). The remaining six retrocopies are like the aforementioned RC_AT5G51410.2 where the absence of sequence hallmarks precludes the inference of the underlying mutational mechanism.

Since the L1 family is widely shared across different plants^17^, we followed the same strategy used in Arabidopsis to identify recently derived retrocopies in another dicotyledonous plant, the cassava, *Manihot esculenta* (*M. esculenta*). We chose cassava because it represents another major branch of dicots that diverged from Arabidopsis more than 100 million years ago^27^. We identified 13 young retrocopies and were able to infer the retroposition mechanism for seven of these on the basis of sequence features: three retrocopies were created by LTR retrotransposons and four were associated with L1-like hallmarks (Supplementary Table S5). For example, R_cassava4.1_019865m harbors a 15-bp polyA tract with an A-to-C substitution, a 13-bp TSD with only 1 mismatch (*E* = 8 × 10^−5^) and an exact copy of the hexanucleotide target sequence “TTAAAA” (Fig. 1C and Supplementary Fig. S11).

## Discussion

By analyzing polymorphic retrocopies and recently evolved retrocopies encoded by reference genomes, we show that in addition to LTR retrotransposons, L1-like machinery also contributes to the formation of retrocopies in dicots. Because L1 retrotransposons are widely shared across dicots and monocots^17^,^18^, it would not be surprising for future work to reveal polymorphic or recently originated retrocopies in monocots with sequence features of L1-like retroposition. The reason that we prefer the term “L1-like” rather than L1 is due to the complexity of the sequence features associated with retroCNVs and retrocopies. Specifically, only R_cassava4.1_019865m (Fig. 1C) fits perfectly with the standard L1-mediated retroposition model in mammals in terms of the polyA tail, TSD and “TTAAAA” target site^2^. In contrast, the other cases show deviations from this model, including highly diverged TSDs, which may be explained by spurious alignments, as suggested by the non-significant BLAST *E*-value and/or non-standard target site found for RC_AT5G58720.1 (Fig. 1A). Certainly, in these cases, L1-like mechanisms may still work, especially considering that these deviations can be explained by mutations subsequent to the retroposition. However, at least for RC_AT5G51410.2 (Supplementary Fig. S7), the L1 or L1-like model hardly works. Even in the relaxed tailless model of L1-mediated retroposition, the TSD and “TTAAAA” remain^14^, whereas the polyA tail, TSD and “TTAAAA” are totally absent for RC_AT5G51410.2. Because this retroCNV is present in only one out of 18 accessions and is thus likely very young (Supplementary Table S1), the absence of all three features is less likely to be accounted for by secondary mutations. It is plausible to think that the retroposition of RC_AT5G51410.2 was mediated by an as yet unknown mechanism. Considering all of these complexities, in-depth experimental work is called for to formally test the functional link between LINE elements and retroposition in plants.

In addition, young retrocopies compiled in this study not only shed light on the underlying retroposition mechanism, but also contribute to future studies on genetic basis of accessions or species-specific phenotypic evolution. For 10 retrocopies encoded by the Arabidopsis reference genome, three are under functional constraint since the ratio between non-synonymous substitution rate and synonymous substitution rate (*Ka/Ks*) relative to parental genes is significantly smaller than 0.5^28^ (Supplementary Table S3). These cases warrant further functional studies.

## Methods

Full-length descriptions are provided in the Supplementary Information.

### Identification and Assembly of RetroCNVs

**Figure 2:**
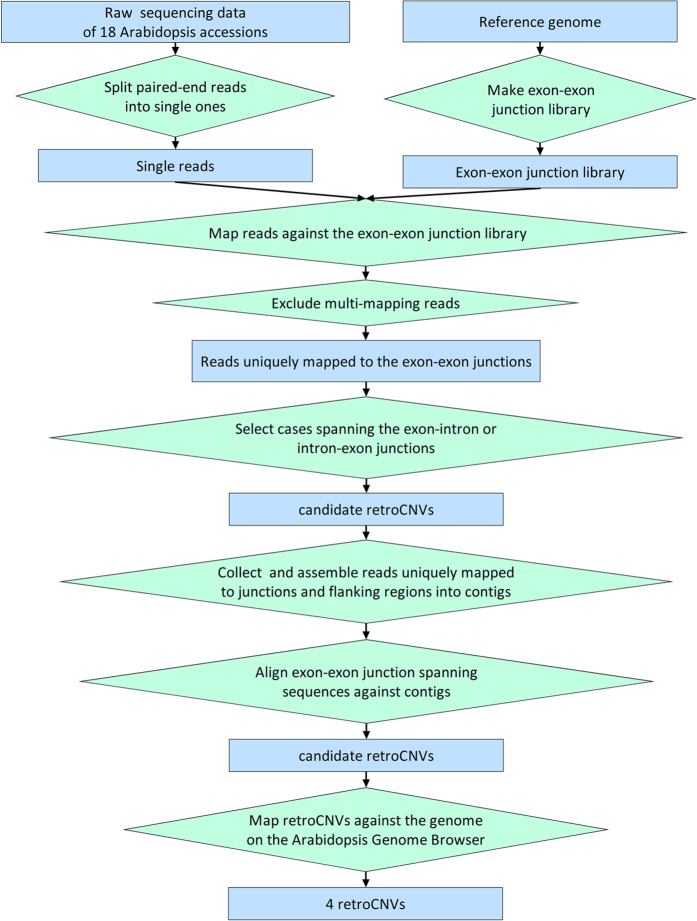
The pipeline for the identification and assembly of retroCNVs.

By improving a previous retroCNV identification strategy^29^,^30^, we identified retroCNVs in Arabidopsis resequencing data^21^ by aligning reads against exon-exon junction sequences and inferring the signal of intron loss (Fig. 2). We then extracted reads that mapped to retroCNVs and performed targeted local *de novo* assembly to obtain information on flanking regions.

### RetroCNV Genotyping in Arabidopsis Accessions

For each of the four retroCNVs, we mapped the assembled longest contig back to the reference genome (TAIR10)^31^,^32^ and determined the insertion site (Supplementary Fig. S12). We then searched for reads with higher alignment quality to retroCNVs than to parental genes and for reads that spanned the insertion breakpoints. If both types of reads were found, we conservatively classified the retroCNV as present in the corresponding accession. Otherwise, the retroCNV was classified as absent.

### LTR/LINE Retrotransposon Inference

To infer the presence of LTR or LINE retrotransposons in the flanking regions of retrocopies, we applied RepeatMasker (http://www.repeatmasker.org) against a customized library that included annotated plant retrotransposons from Repbase^33^,^34^ and TIGR^35^, as well as retrotransposons predicted *de novo* via MGESCan-LTR^36^ and MGEScan-nonLTR^37^.

### Identification of Newly Evolved Retrocopies in Dicot Reference Genomes

We implemented BLAT^38^ and aligned the mRNAs derived from genes with at least one intron against the reference genomes of Arabidopsis and cassava (version 4.1)^39^. We then processed the alignment information and inferred the candidate retrocopy by retaining consecutive hits (BLAT identity higher than 95%), suggesting an intron loss event.

## Additional Information

**How to cite this article**: Zhu, Z. *et al*. LINE-1-like retrotransposons contribute to RNA-based gene duplication in dicots. *Sci. Rep*. **6**, 24755; doi: 10.1038/srep24755 (2016).

## Supplementary Material

Supplementary Information

## Figures and Tables

**Table 1:** RetroCNVs in Arabidopsis.

PG	AS	E-E	E-I	I-E	IS	N	Flank	PolyA	TSD	TTAAAA	Mechanism
AT3G06040.1	Can-0	7	17	14	Chr2(+): 7.9M	3		N	N	Y	Uncertain
AT3G08580.2	No-0	25	22	25	Chr2(−): 11.7M	14	LTR/LTR	N	N	N	LTR
AT5G58720.1	Oy-0	108	73	72	Chr4(+): 7.6M	3		Y	Y	Y	L1-like
AT5G51410.2	No-0	26	18	26	Chr1(+): 12.3M	1		N	N	N	Uncertain

“PG” means the parental gene from which the retroCNV is derived. Since parental gene could encode multiple isoforms, only one transcript model (“0.1” or “0.2”) with the highest sequence similarity to the retroCNV is listed. “AS” denotes the accession in which the retroCNV is assembled, while “N” denotes the number of accessions in which the retroCNV is present. In the columns entitled “E-E” (reads mapped to exon-exon junctions), “E-I” (reads mapped to exon-intron junctions) or “I-E” (reads mapped to intron-exon junctions), the numbers refer to the total count of reads mapped to the corresponding junctions. “IS” is the coordinate of the insertion site with “+/−” showing the orientation of the retroCNV relative to the sense strand of the inserted chromosome (Chr). “Flank” shows the retrotransposons in the 5′/3′ flanking region of the retroCNV. Only the retroCNV derived from AT3G08580.2 is flanked by LTR retrotransposon at both sides whereas no recognizable retrotransposon is associated with the other three cases. The next three columns “PolyA”, “TSD” and “TTAAAA” list whether there is polyA, TSD or TTAAAA-like sequences in the flanking region, respectively. Given these sequence features, the mechanism is inferred in the last column.

## References

[b1] Chen S., Krinsky B. H. & Long M. New genes as drivers of phenotypic evolution. Nat Rev Genet 14, 645–660 (2013). 23949544 10.1038/nrg3521 PMC4236023

[b2] Kaessmann H., Vinckenbosch N. & Long M. RNA-based gene duplication: mechanistic and evolutionary insights. Nat Rev Genet 10, 19–31 (2009). 19030023 10.1038/nrg2487 PMC3690669

[b3] Katju V. In with the old, in with the new: the promiscuity of the duplication process engenders diverse pathways for novel gene creation. Int J Evol Biol 2012, 341932 (2012). 23008799 10.1155/2012/341932 PMC3449122

[b4] Long M. & Langley C. H. Natural selection and the origin of jingwei, a chimeric processed functional gene in Drosophila. Science 260, 91–95 (1993). 7682012 10.1126/science.7682012

[b5] Wang W. . High rate of chimeric gene origination by retroposition in plant genomes. Plant Cell 18, 1791–1802 (2006). 16829590 10.1105/tpc.106.041905 PMC1533979

[b6] Zhu Z., Zhang Y. & Long M. Extensive structural renovation of retrogenes in the evolution of the Populus genome. Plant Physiol 151, 1943–1951 (2009). 19789289 10.1104/pp.109.142984 PMC2785971

[b7] Abdelsamad A. & Pecinka A. Pollen-specific activation of Arabidopsis retrogenes is associated with global transcriptional reprogramming. Plant Cell 26, 3299–3313 (2014). 25118244 10.1105/tpc.114.126011 PMC4371830

[b8] Xiao H., Jiang N., Schaffner E., Stockinger E. J. & van der Knaap E. A retrotransposon-mediated gene duplication underlies morphological variation of tomato fruit. Science 319, 1527–1530 (2008). 18339939 10.1126/science.1153040

[b9] Matsuno M. . Evolution of a novel phenolic pathway for pollen development. Science 325, 1688–1692 (2009). 19779199 10.1126/science.1174095

[b10] Wicker T. . A unified classification system for eukaryotic transposable elements. Nat Rev Genet 8, 973–982 (2007). 17984973 10.1038/nrg2165

[b11] Esnault C., Maestre J. & Heidmann T. Human LINE retrotransposons generate processed pseudogenes. Nat Genet 24, 363–367 (2000). 10742098 10.1038/74184

[b12] Rawal K. & Ramaswamy R. Genome-wide analysis of mobile genetic element insertion sites. Nucleic Acids Res 39, 6864–6878 (2011). 21609951 10.1093/nar/gkr337 PMC3167599

[b13] Richardson S. R., Morell S. & Faulkner G. J. L1 retrotransposons and somatic mosaicism in the brain. Annu Rev Genet 48, 1–27 (2014). 25036377 10.1146/annurev-genet-120213-092412

[b14] Noll A., Raabe C. A., Churakov G., Brosius J. & Schmitz J. Ancient traces of tailless retropseudogenes in therian genomes. Genome Biol Evol 7, 889–900 (2015). 25724209 10.1093/gbe/evv040 PMC5322556

[b15] Jin Y. K. & Bennetzen J. L. Integration and nonrandom mutation of a plasma membrane proton ATPase gene fragment within the Bs1 retroelement of maize. Plant Cell 6, 1177–1186 (1994). 7919987 10.1105/tpc.6.8.1177 PMC160511

[b16] Elrouby N. & Bureau T. E. Bs1, a new chimeric gene formed by retrotransposon-mediated exon shuffling in maize. Plant Physiol 153, 1413–1424 (2010). 20488894 10.1104/pp.110.157420 PMC2899935

[b17] Smyshlyaev G., Voigt F., Blinov A., Barabas O. & Novikova O. Acquisition of an Archaea-like ribonuclease H domain by plant L1 retrotransposons supports modular evolution. Proc Natl Acad Sci USA 110, 20140–20145 (2013). 24277848 10.1073/pnas.1310958110 PMC3864347

[b18] Ohshima K. RNA-Mediated Gene Duplication and Retroposons: Retrogenes, LINEs, SINEs, and Sequence Specificity. Int J Evol Biol 2013, 424726 (2013). 23984183 10.1155/2013/424726 PMC3747384

[b19] Zhang X. & Wessler S. R. Genome-wide comparative analysis of the transposable elements in the related species Arabidopsis thaliana and Brassica oleracea. Proc Natl Acad Sci USA 101, 5589–5594 (2004). 15064405 10.1073/pnas.0401243101 PMC397431

[b20] Zhang Y., Wu Y., Liu Y. & Han B. Computational identification of 69 retroposons in Arabidopsis. Plant Physiol 138, 935–948 (2005). 15923328 10.1104/pp.105.060244 PMC1150409

[b21] Gan X. . Multiple reference genomes and transcriptomes for Arabidopsis thaliana. Nature 477, 419–423 (2011). 21874022 10.1038/nature10414 PMC4856438

[b22] Dai H., Yoshimatsu T. F. & Long M. Retrogene movement within- and between-chromosomes in the evolution of Drosophila genomes. Gene 385, 96–102 (2006). 17101240 10.1016/j.gene.2006.04.033

[b23] Zhou Q. . On the origin of new genes in Drosophila. Genome Res 18, 1446–1455 (2008). 18550802 10.1101/gr.076588.108 PMC2527705

[b24] Tan S., Zhu Z., Zhu T., Te R. & Zhang Y. E. Chance and Necessity: Emerging Introns in Intronless Retrogenes. eLS doi: 10.1002/9780470015902.a0022886 (2014).

[b25] Zhang C., Gschwend A. R., Ouyang Y. & Long M. Evolution of gene structural complexity: an alternative-splicing-based model accounts for intron-containing retrogenes. Plant Physiol 165, 412–423 (2014). 24520158 10.1104/pp.113.231696 PMC4012599

[b26] Ma J., Devos K. M. & Bennetzen J. L. Analyses of LTR-retrotransposon structures reveal recent and rapid genomic DNA loss in rice. Genome Res 14, 860–869 (2004). 15078861 10.1101/gr.1466204 PMC479113

[b27] Hedges S. B., Dudley J. & Kumar S. TimeTree: a public knowledge-base of divergence times among organisms. Bioinformatics 22, 2971–2972 (2006). 17021158 10.1093/bioinformatics/btl505

[b28] Betran E., Thornton K. & Long M. Retroposed new genes out of the X in Drosophila. Genome Res 12, 1854–1859 (2002). 12466289 10.1101/gr.604902 PMC187566

[b29] Schrider D. R., Stevens K., Cardeno C. M., Langley C. H. & Hahn M. W. Genome-wide analysis of retrogene polymorphisms in Drosophila melanogaster. Genome Res 21, 2087–2095 (2011). 22135405 10.1101/gr.116434.110 PMC3227099

[b30] Schrider D. R. . Gene copy-number polymorphism caused by retrotransposition in humans. Plos Genet 9, e1003242 (2013). 23359205 10.1371/journal.pgen.1003242 PMC3554589

[b31] Huala E. . The Arabidopsis Information Resource (TAIR): a comprehensive database and web-based information retrieval, analysis, and visualization system for a model plant. Nucleic Acids Res 29, 102–105 (2001). 11125061 10.1093/nar/29.1.102 PMC29827

[b32] Poole R. L. The TAIR database. Methods Mol Biol 406, 179–212 (2007). 18287693 10.1007/978-1-59745-535-0_8

[b33] Jurka J. Repbase update: a database and an electronic journal of repetitive elements. Trends Genet 16, 418–420 (2000). 10973072 10.1016/s0168-9525(00)02093-x

[b34] Jurka J. . Repbase Update, a database of eukaryotic repetitive elements. Cytogenet Genome Res 110, 462–467 (2005). 16093699 10.1159/000084979

[b35] Ouyang S. & Buell C. R. The TIGR Plant Repeat Databases: a collective resource for the identification of repetitive sequences in plants. Nucleic Acids Res 32, D360–363 (2004). 14681434 10.1093/nar/gkh099 PMC308833

[b36] Rho M., Choi J. H., Kim S., Lynch M. & Tang H. *De novo* identification of LTR retrotransposons in eukaryotic genomes. BMC Genomics 8, 90 (2007). 17407597 10.1186/1471-2164-8-90 PMC1858694

[b37] Rho M. & Tang H. MGEScan-non-LTR: computational identification and classification of autonomous non-LTR retrotransposons in eukaryotic genomes. Nucleic Acids Res 37, e143 (2009). 19762481 10.1093/nar/gkp752 PMC2790886

[b38] Kent W. J. BLAT–the BLAST-like alignment tool. Genome Res 12, 656–664 (2002). 11932250 10.1101/gr.229202 PMC187518

[b39] Prochnik S. . The Cassava Genome: Current Progress, Future Directions. Trop Plant Biol 5, 88–94 (2012). 22523606 10.1007/s12042-011-9088-z PMC3322327

