# Supplementary material for: LINE-1-like retrotransposons contribute to RNA-based gene duplication in dicots
Source: Sci Rep. 2016 Apr 21;6:24755. doi: 10.1038/srep24755 (PMC4838847; doi:10.1038/srep24755)
Supplement: Supplementary Information [file srep24755-s1.pdf]

Supplementary Information

LINE-1-like retrotransposons contribute to RNA-based gene duplication in dicots

Zhenglin Zhu[1], Shengjun Tan[2], Yaqiong Zhang[2], Yong E. Zhang[2,3]

1. School of Life Sciences, Chongqing University, Chongqing 400044, China

2. Key Laboratory of the Zoological Systematics and Evolution & State Key Laboratory of Integrated Management of Pest Insects and Rodents, Institute of Zoology, Chinese Academy of Sciences, Beijing 100101, China

3. University of Chinese Academy of Sciences, Beijing 100049, China

This file consists of full materials and methods, supplementary tables and supplementary figures.

Index

Methods....3
- Identification and assembly of retroCNVs....3
- RetroCNV genotyping in Arabidopsis accessions....4
- LTR/LINE retrotransposon inference....5
- Identification of newly evolved retrocopies in dicot reference genomes....6

Supplementary Tables....7
- Table S1....7
- Table S2....8
- Table S3....9
- Table S4....11
- Table S5....12

Supplementary Figures....13
- Figure S1....18
- Figure S2....22
- Figure S3....26
- Figure S4....27
- Figure S5....30
- Figure S6....31
- Figure S7....33
- Figure S8....35
- Figure S9....37
- Figure S10....38
- Figure S11....39
- Figure S12....40

References in the Supplementary Information....41

Methods

Identification and assembly of retroCNVs

We identified retroCNVs in Arabidopsis (*Arabidopsis thaliana*) by integrating several published retroCNV identification strategies (Fig. 2)[1,2]. Specifically, we downloaded the fastq-format Illumina sequencing data of 18 Arabidopsis accessions, with sequencing coverage ranging from 27-fold to 36-fold and read length ranging from 36 to 51 bp[3]. We split all paired-end reads into single reads to retain cases of retroposition in which one read is mapped to the parental locus and its partner is mapped to the insertion site. We also retrieved the reference genome and gene annotations from TAIR10[4,5]. We mapped reads against the exon-exon junction library (200 bp) using NovoAlign (version 2.08, www.novocraft.com) with the parameters "-o Softclip -r All 10 -s 1", which allows up to the top 10 alignment hits for one read and enables automatic trimming at two ends. For reads with more than one hit, we kept reads with differences in their alignment scores of greater than 5 and with identities greater than 5% between the top 1 and 2 alignment positions. In this way, we excluded all multi-mapping reads, *i.e.*, reads that mapped to more than one location equally well. Then, we pulled out reads that uniquely mapped to the exon-exon junctions after removing reads generated by PCR duplication using Picard (picard.sourceforge.net). We called an event retroposition or intron loss if there were at least three reads from one accession spanning the exon-exon junctions with an overhang (⩾10 bp). The introns of parental genes are present during retroposition but are absent during intron loss. Thus, in the case of retroposition, we expected to detect reads that mapped to both exon-intron and intron-exon junctions, suggesting the existence of parental introns. On this basis, we further selected cases in which at least three reads spanned the exon-intron or intron-exon junctions with an overhang (⩾10 bp). After applying these two filtering steps, we identified candidate retroCNVs in Arabidopsis accessions.

In contrast to previous efforts[1,2], we next performed targeted *de novo* assembly. We

collected reads that were uniquely mapped to exon-exon junctions and the 500-bp flanking regions of the parental genes. We assembled these reads using MIRA[6], which is able to recognize differences between parental and retro copies, including SNPs and intron deletions. Afterwards, we retrieved exon-exon junction-spanning sequences with 20 bp on each side and used BLAT[7] to align these 40-bp sequences against contigs assembled by MIRA. Given the output, we further extracted candidate retroCNVs that covered the junctions with both identity and coverage higher than 95%. Then, we mapped all retroCNVs against the genome using BLAT on the Arabidopsis Genome Browser (epigenomics.mcdb.ucla.edu) and retained only those cases with the hallmark of intron loss compared with the presumed parental gene. Finally, for these retroCNVs, we implemented the PRICE package[8] to extend the flanking regions by searching and merging reads aligned to the contigs generated by MIRA. We were able to assemble the flanking regions of four retroCNVs. For each retroCNV, we took the longest contig (median length, 528 bp) in one accession as the template for downstream mechanistic analyses. All the reads mapping to retroCNVs and their insert sites, especially those spanning exon-exon junctions, and the breaking points between retroCNVs and flanking regions are shown in Fig. S2, S3, S5 and S7 (Panels D-F).

RetroCNV genotyping in Arabidopsis accessions

Next, we employed a conservative approach to determine the presence/absence of the four retroCNVs across the 18 accessions (Fig. S12). Specifically, we mapped the reads of the different accessions onto the reference genome as well as the template retroCNVs using NovoAlign (www.novocraft.com) with the aforementioned parameters "-r All 10 -s 1". We extracted the reads that were uniquely mapped to retroCNVs and used BLAT[7] to align these reads against the retroCNVs and the corresponding parental genes. We retained reads that mapped to the retroCNVs with at least 95% coverage and that had higher identity than when mapped to the parental genes. If at least two reads mapped better to the retroCNVs, we again assembled these reads into longer contigs using MIRA[6]. We then searched the contigs against the

retroCNVs and the reference genome using BLAT[7]. If the retroCNV produced a better alignment, we classified it as presence in this accession.

In parallel, given that the 3' breakpoint may be disturbed by polyA sequences, we extended the 5' breakpoint of the retroCNV of interest by 50 bp and used the spanning 100-bp sequence to search reads in the 18 accessions. We then assembled the mapped reads using MIRA[6]. If we generated a contig that spanned the 5' breakpoint, we classified this as the presence of the insertion site.

Overall, we conservatively assigned the presence of retroCNVs across accessions by requiring both the presence of the retroposed region and the corresponding 5' breakpoint sequences. In other words, the population frequency in Table S1 represents a lower-bound estimation.

LTR/LINE retrotransposon inference

To infer whether the flanking regions of retroCNVs and recently evolved retrocopies encoded by the reference genomes consist of LTR/LINE retrotransposons, we used RepeatMasker (www.repeatmasker.org) to search a customized repeat library. Considering the issue of incomplete annotation, this library not only included known plant retrotransposons listed in Repbase[9,10] and TIGR[11] but also covered retrotransposons predicted in the reference genomes of Arabidopsis and the cassava, *Manihot esculenta* (*M. esculenta*) (version 4.1)[12] by two *de novo* strategies based on MGESCan-LTR [13] and MGEScan-nonLTR[14]. We clustered all repeat elements via CD-HIT[15], with an identity cutoff of 80%, considering that in MGESCan-LTR, the identity cutoff of the upstream and downstream long terminal repeat was set at 80%[13]. Based on our comprehensive and non-redundant repeat library, we scanned for repeat elements using RepeatMasker with the parameter "-s" to increase the sensitivity. Given that the retroelement structure could rapidly degenerate[16], we specified relatively relaxed criteria: coverage > 50% or mapped length > 150 bp, divergence < 50% and SW score > 250.

Identification of newly evolved retrocopies in dicot reference genomes

As in the case of retroCNVs, we again searched for the hallmark of intron loss and identified retrocopies encoded by Arabidopsis and the *M. esculenta* (version 4.1). For both species, we first extracted exon-exon junction sequences by extending 20 bp beyond the junction. Then, we aligned these sequences against the reference genome via BLAT. We extracted the possible segments of candidate retrocopies by only retaining alignments with a single block, suggesting an intron loss event. Moreover, because we were interested in recently evolved retrocopies, we required that the alignment showed high identity (⩾95%) and high coverage (⩾95%). Given these short alignments indicating intron loss, we further extended the boundaries of each candidate retrocopy and its corresponding parental gene by 1,000 bp and aligned them using BLAT. We reiterated this step until we could no longer extend the alignment. After this step, we inferred the breakpoint of the retrocopy and the insertion site.

For all candidate retrocopies, we performed the following two filters. First, there were cases with one parental gene and multiple paralogous retrocopies that may represent secondary DNA-level duplications of the first retrocopy. Thus, we only kept the retrocopy with the highest similarity to the parental gene (*i.e.,* the one with the highest BLAT score). Second, we manually checked the candidate retrocopies by mapping them onto the reference genome using BLAT on the Arabidopsis Genome Browser (epigenomics.mcdb.ucla.edu) and only retained the entries that showed spliced alignment against the corresponding parental genes. We genotyped 10 recently evolved retrocopies in the 18 Arabidopsis accessions by searching these retrocopies in the assembled genomes of the 18 accessions[3] via BLAT[7].

Supplementary Tables

Table S1. Genotyping of retroCNVs across 18 accessions.

The name of each retroCNV is represented by "RC_" (short for retroCNV) followed by its parental gene accession. "Y" denotes presence, whereas "N" denotes absence.

RetroCNV	Bur-0	Can-0	Ct-1	Edi-0	Hi-0	Kn-0	Ler-0	Mt-0	No-0	Oy-0	Po-0	Rsch-4	Sf-2	Tsu-0	Wil-2	Ws-0	Wu-0	Zu-0
RC_AT3G06040.1	N	Y	N	N	Y	N	N	N	N	N	N	N	Y	N	N	N	N	N
RC_AT3G08580.2	N	N	N	Y	Y	Y	N	Y	Y	Y	Y	Y	Y	Y	Y	Y	Y	Y
RC_AT5G58720.1	N	N	N	N	N	N	N	N	N	Y	Y	N	N	N	N	N	N	Y
RC_AT5G51410.1	N	N	N	N	N	N	N	N	Y	N	N	N	N	N	N	N	N	N

Table S2. The length (bp) of the assembled retroCNVs and their flanking regions.

RetroCNV	Retrocopy	5' flanking region	3' flanking region
RC_AT3G06040.1	627	597	1,321
RC_AT3G08580.2	1,019	556	27
RC_AT5G58720.1	418	500	661
RC_AT5G51410.2	60	488	500

Table S3. Newly evolved retrocopies encoded by the Arabidopsis reference genome.

The convention largely follows Table 1 in the main text. "Reported" denotes whether the retrocopy is identified previously[17-19]. Only the retrocopy derived from the parental gene AT5G37150.1 is associated with LTR retrotransposon at two sides. Another three cases associated with a polyA tail but not LTR retrotransposons were possibly driven by an L1-like mechanism. The remaining six cases are associated with either a previously undescribed mechanism or with an LTR or L1-like mechanism in which the sequence signatures have already degenerated over evolutionary time. Thus, we called these cases "uncertain" in the last column. After aligning the sequences between the retrocopy and its corresponding parental gene on the basis of reading frame of the later, we calculated non-synonymous substitution rate (*Ka*) and synonymous substitution rate (*Ks*) via the codeml program in the PAML package[20]. We then performed the likelihood ratio test on whether *Ka/Ks* is significantly smaller than 0.5[21]. Such a conservative criteria ensured that the retrocopy must be under constraint even the parental locus is neutrally evolving with a *Ka/Ks* of 1.

PG	Locations	Reported	Flank	PolyA	TSD	TTAAAA	Mechanism	*Ka*	*Ks*	*Ka/Ks*	*P*-value
AT1G05890.1	Chr1(+): 23655643-23657487	Y		Y	N	N	L1-like	0.058	0.087	0.662	0.356
AT1G17780.2	Chr2(+): 7184534-7185341	N		N	N	N	uncertain	0.045	0.107	0.420	0.436
AT1G50010.1	Chr4(-): 8548602-8550668	N		N	N	N	uncertain	0.002	0.297	0.007	0.000
AT1G60170.1	Chr3(-): 22403267-22404967	Y		N	N	N	uncertain	0.000	0.000	NA	0.954
AT2G45330.1	Chr5(+): 7956005-7956655	Y		N	N	N	uncertain	0.021	0.094	0.224	0.030
AT3G23100.1	Chr1(-): 22658630-22659475	Y		N	N	N	uncertain	0.028	0.161	0.176	0.003
AT4G01590.1	Chr4(+): 16919126-16919798	Y		N	N	N	uncertain	0.060	0.144	0.415	0.491
AT4G21660.1	Chr1(+): 3873203-3873861	N		Y	N	N	L1-like	0.031	0.028	1.116	0.270
AT4G31900.1	Chr4(-): 7356209-7359090	N		Y	Y	N	L1-like	0.138	0.102	1.355	0.037
AT5G37150.1	Chr5(-): 21167349-21169533	Y	LTR/LTR	N	N	N	LTR	0.0171	0.0516	0.332	0.090

Table S4. Genotyping of recently evolved retrocopies in the 18 accessions.

The name of each retrocopy is represented by "R_" (short for recently evolved retrocopy) and its parental gene. "Y" denotes presence, whereas "N" denotes absence.

Retrocopy	Bur-0	Can-0	Ct-1	Edi-0	Hi-0	Kn-0	Ler-0	Mt-0	No-0	Oy-0	Po-0	Rsch-4	Sf-2	Tsu-0	Wil-2	Ws-0	Wu-0	Zu-0
R_AT1G05890.1	Y	Y	Y	Y	Y	Y	Y	Y	Y	Y	Y	Y	Y	Y	Y	Y	Y	Y
R_AT1G17780.2	Y	Y	Y	Y	Y	Y	Y	Y	Y	Y	Y	Y	Y	Y	Y	Y	Y	Y
R_AT1G50010.1	Y	Y	Y	Y	Y	Y	Y	Y	Y	Y	Y	Y	Y	Y	Y	Y	Y	Y
R_AT1G60170.2	Y	Y	Y	Y	Y	Y	Y	Y	Y	Y	Y	Y	Y	Y	Y	Y	Y	Y
R_AT2G45330.1	Y	Y	Y	Y	Y	Y	Y	Y	Y	Y	Y	Y	Y	Y	Y	Y	Y	Y
R_AT3G23100.1	Y	Y	Y	Y	Y	Y	Y	Y	Y	Y	Y	Y	N	Y	Y	Y	Y	Y
R_AT4G01590.1	Y	Y	Y	Y	Y	Y	Y	Y	Y	Y	Y	Y	Y	Y	Y	Y	Y	Y
R_AT4G21660.1	Y	Y	Y	Y	Y	Y	Y	Y	Y	Y	Y	Y	Y	Y	Y	Y	Y	Y
R_AT4G31900.1	Y	Y	Y	Y	Y	Y	Y	Y	Y	Y	Y	Y	Y	Y	Y	Y	Y	Y
R_AT5G37150.1	Y	Y	Y	Y	Y	Y	Y	Y	Y	Y	Y	Y	Y	Y	Y	Y	N	Y

Table S5. Newly evolved retrocopies encoded by the *M. esculenta* reference genome.

The convention follows Table 1. LTR retrotransposons could be identified in only one side for three cases. Another four cases associated with the polyA tail but not LTR retrotransposons were possibly driven by an L1-like mechanism. The remaining six cases are associated with either an undescribed mechanism or with an LTR or L1-like mechanism in which the sequence signatures have already degenerated over evolutionary time.

Retrocopy	Parental gene	Location	Flank	PolyA	TSD	TTAAAA	Mechinism
R_cassava4.1_000716m	*cassava4.1_000716m*	scaffold12341(+): 19473-21103	/LTR	N	N	N	LTR
R_cassava4.1_000867m	*cassava4.1_000867m*	scaffold02865(+): 83962-85172	LTR/	N	N	N	LTR
R_cassava4.1_001372m	*cassava4.1_001372m*	scaffold12746(-): 404-1682		N	N	N	uncertain
R_cassava4.1_002584m	*cassava4.1_002584m*	scaffold02658(-): 984514-985182		N	N	N	uncertain
R_cassava4.1_006936m	cassava4.1_006936m	scaffold07238(+): 538800-540305		Y	Y	Y	L1-like
R_cassava4.1_007181m	cassava4.1_007181m	scaffold06700(+): 141611-141969	LTR/	N	N	N	LTR
R_cassava4.1_012117m	cassava4.1_012117m	scaffold06582(-): 853091-854334		Y	N	N	L1-like
R_cassava4.1_012226m	cassava4.1_012226m	scaffold00325(-): 525590-526615		N	N	N	uncertain
R_cassava4.1_015105m	cassava4.1_015105m	scaffold07329(-): 35740-36636		Y	N	N	L1-like
R_cassava4.1_018827m	cassava4.1_018827m	scaffold00847(-): 1619684-1620083		N	N	N	uncertain
R_cassava4.1_019865m	cassava4.1_019865m	scaffold08617(-): 71499-72052		Y	Y	Y	L1-like
R_cassava4.1_019883m	cassava4.1_019883m	scaffold00847(-): 1619720-1620130		N	N	N	uncertain
R_cassava4.1_033677m	cassava4.1_033677m	scaffold10563(-): 1366705-1367705		N	N	N	uncertain

Supplementary Figures

A

TCCGTTATCTCCGCCGAACCATCCATCCTCCGCTACTTCATCTCCGCCGCTGAGATCGGAATCAC
TATCCCACTCCATTTGATTTTGCATACACACCCACAAAATAAAGCTTAAGACTGCCCACGAATCT
TCTTCCTCAGCGACAGGAGAAGAGACCCAGAAGAACACAGTTTGATTTTGAATCGCGGAATCTGA
TATCTGTAGGTAATCGAAGTCTCCACAGGAAAAAGTTCAAAACTTTAGACAAACCCAGAAATCGT
CTTCTTCAAGAACAGGAGACAGAAGAACAGATTTTGAAATGGAATCAAGCGAAGGAAAGAGGAAT
CTGATATTTGTAGGTAATGGAAGTCTCCACCGCCCACTTCTAAGAGGAGGAGAAGAAGAAGAGAG
ATGATTCGGTCGCTCGATGACTCGGCTCTTCTCAGTGCCTCTCCTTTTCCATCTTTAACCAGACC
GGTTTCTTAATTTTACCTTACCGGTTTAGTTTATTTATCCGGTTTACACAAAATATCCTGTAAGT
TCCTTGTGAGATTTTTTTGGTTTTACCAAATGAGTGTATTAGAATCAATTTTAATGATTTAGAAA
CATTAGAAACATCGTGACTCAGCTCCGTCTCTCACTGTAATAATCAAGTCAGAGCCGACGAAGTT
GACGTTTGCGCCGTCGGAGGAGAGTTTTTACTGTTGCTGTCCAGTTGACATTTTGAGACATGAAA
TGATCTGGGGTTGATGTTTGTATGGTAACAGAGGAATTATAGTCATGAAGCTTATTTCACTTGTC
AGAAACGTTCGTTCTCGCCAATGTCAACCGGAAGTTATCTGGTCTTTGCAAGTTCGTTTCTTGCA
GCAAGATTCTGTCTCGAAAGCTAAACCCAAGAAATACAAACACCCGTCAGTTTATGATCCGTATG
GTCCTAGACCCCAGCCTTCAAGCAAAATCATGGAGCTAGCTGAGCGTATAGCTGCATTATCTCCA
GAAGAAAGAAAACAGATTGGTCCTGCTCTCAATGAACACCTGAGGCTTCCAAAACAACAGATGAT
TTCATCGGACGGCATTGGAGCAAACAAGATACGGAGCTGGGAATGTAGAGGAGAAGAAGGAGAAG
ACGGCTTTCGATGTGAAGTTGGAGAAGTTTAATGCATCTGATAAGATCAAAGTGATAAAAGAAGT
TAGAACGTTCACAAGTTTGGGTCTGAAGGAAGCGAAAGAGCTTGTGGAGAAAGGATAATAGTCTT
TTTCTACTTTCGATCTCAAAAACTACTAAAAAGTGAAGCCTTGTAAATCTTCTTTAAAGAGTAGA
AACATGTATTATTATCTTTTAGTTATTCATCTTCAAAGTTTTGATTAATTTAAAGTGAATGAAAT
ATAAATTCAATACAAAAAAAGAAGAAACCCAAATCTACAAAAGAAAAAAAGAAAAATATATAATT
GATTTAGGATTCTAAATTCTCACGTACTCGGAGAGCAAGCCGTTGAATAGACTGATCATCGAAGC
AAGGATCAAAGGTCTTAATCTTGGACACAAAATCCATAGCTTCACTATACTTCCCTGCTCTGCAA
TAACCATCTACTACCATTTTAAAAGTCAGCTCATTTGGTCTGCAATCATTCTTCGCCATGCACTC
AATCACATCTTCTATCTCTGCAAACATTCCCATCGCCGTGTAACCCGAAACAAACGTGTTGTAAG
TGAAAATGCACGGTCTGATTCCCCGCTCTGTCATCTCAGACAGCATCCTAACCGCTTCTTGCATC
AACCCTCTTCTGCAGAAACCTTTGATCACTGTGTTGTAAGAGACCAGGTCCGGTTTTAACTGCGA
TTTTTCTAGAGTCTTGAGGATTTCTTCGGCTTTCCAACACTCTCCTCTTCTTACGTACATGTCCA
TCAGG

B

TTATATGATAACATTCCTAAATTTTCAAAGGTGTCTATCAATCTATAAAACCATGATTACTTTCT
GAAACCTTCCAGAAATTTACTAAAAACATGTTGTTCCTAGATACTTTTGAGTTTTGACACAATGA
GGACCATTCCTATATCTTTTGTAAGATACTCTAGTAGTCTAGTCCTACTGTCTCGATCTTCTATT
AAATCTTTAAGTATAGAAGATCCAAGTAAACTTACATATAGTTAATAGAAGATCCAAGTAAGATC
AAAGCCAATAATATTAAATTTATATAAAAAAAAATTGTAAACACTGTTTTTTTTTTCAATTGATG
AAAGGAAAAAAGCATTCAGGAATTGTTTTTATTTTTATTTTTTTGTTAACATTCATTTATTTAAA
TTGCATAAGAAAAGAAAAAAGTGATAACCATTGCCAGAAACAAAGGTATAGAGAATGTGGTAACC
ATTGATCTGCATGGTCAGCATGTTAAACCAGCAATGAAGCTACTGAAGCTACATCTGTTATTTGG
ATCATATGTTCCAGCCATTCAGACTCTACGAGTGATCACAGGATGTGGAGCTTCTGGGTTTGGGA
AGTCTAAGGTGAAACAATCAGTGGTAAAGCTGCTAGAAAGAGAAGGAGTTAGGTATTGTGAAGAG
AACAGAGGGACACTGCTGATCAAGCTTGACGGAGGTAGTAGAGAGTTCAGTTTCTTAGACACAGA
GAGTGACTCTGATGAATAAGTGATAACTAAAACTAAAGTCAGGTTTTAGCTTTAGATCTTAAAAT
TTATGTCGATTTTGCCTATATCTGATGCTAGCTCTCTGTTGTTAAGTAAATGTTGAGCAAAAAAA
AAAAAAAGTTAACAAGCCTTAGACAAAAAATTAAGAGCCCAATAACGAAAGTTGAACTGTAAAGA
AACGAAATATAACTAGTTGTAGAATTGTATATATAGGATAGCTAGTAAAAAAGAGTGGTTGTTCT
GTAACTACAATCATTTATTTTTTTGTTGACATTCGTAATCATTGTAACATACGA

C

AATGTTGGCGACCCACAACCCCAATGCGTTTCCAACACATTCAAAAGCTGACAGTTTGACAAATT
GTGAAAGGTCACATCAACTTGACAAAGAGATTCATTTCCTTTGCCACTTTTGAATTATTTATATG
TAATTTCTTTCCTTTTCCATAAAAAAAAACTATGAGGTAGAAAAACAAGATCTGGGTTTCCTCTT
ATAGAAACCCTGATTCCAACCTAACTGTCTTCAGATTACACGTTTCAAGTGTACTTTGCATGTGA
TCGTGATAATATTTTTAATTATTATTTTTTTATTTTAGCATATGCTCCCACAACAATCTGTGACA
CTATCACACTAACATTAATATTAAAAGCACAACATGTCAATCATATATACGGTCTTATTCGCAAG
TCCTGTGATAGAACTTTTCTTCTCAGGTAGAGGAAACCAACCTTTCATTTGTTCCTTGAAACTGA
AAAGTAAAAAAACAATCGAAATTAAAGCTCGTCAGTGTTACATCGTTTAATTAGAGCCGTTATTC
GTGACTTACACTGATACCATATTAGAGTGTGGGCTTCCAACCTAAAACCAATTGGCAATAGGTGG
AGAGGCCCATATCTTATATATACCACTTAAGATCCTACTCAACTTCCGATGTGGGACATTGTCCC
TAATACGCCCCCTCGAGATGATGGCTCTTCTAGCCATTGATCTCGATATGTTTGGGCATGGATCG
GCGGGCCAAGTATTGGGCCGGACCGATGTGGATCGGGTTGAGTATGTGCGGATCGGGCTCTGATA
CCATATTAGACTGTGGGCTTCTACACCACAGAAAAAACTTCGTCTCTCTTCTCTGCTTCGCCCTC
TCATTTCCTGTGAGATAAAGGCGGAGTCTCTCTCCAATTATTTTGCTCATCCATCGATTCTTAGA
GTTCAAAATGGTTGATCAAGTTCAGCACCCCACTATTGCGCAGAAAGCTGCCGGGCAGTTCATGC
GTTCAAGTGTTTCCAAGGACGTTCAAGTGGGTTACCAGAGGCCTTCTATGTATCAAAGACATGCA
ACCTACGGAAACTACTCCAATGCTGCATTTCAATTTCCTCCCACATAGGAGAGAAGGGGTTCACT
AACTTTGCCCTTGACTTTCTGATGGGTGGTGTTTCTGCTGCCGTCTCCAAGACTGCTGCTGCTCC
TATTGAACGTGTTAAGCTTTTGATCCAGAACCAGGATGAGATGATTAAAGCTGGCAGGCTTTCTG
AACCCTACAAGGGTATTGGTGACTGTTTCGGCAGGACGATTAAGGATGAAGGTTTTGGTTCTCTA
TGGAGAGGCAACACTGCCAATGTTATCCGTTATTTCCCCACTCAGGTTTGTTGAGTTTCATACTC
TTTCTTGTTATAGCTTTTGAAAAAACATAATTTTGTGCTAACCTTCTTTTTTGTCTATTGTAGGC
CTTGAACTTTGCCTTCAAAGATTACTTCAAAAGACTTTTCAACTTTAAGAAGGACAGAGATGGTT
ACTGGAAGTGGTTTGCTGGTAACTTGGCATCTGGAGGAGCAGCTGGTGCCTCTTCCCTTCTGTTT
GTGTACTCCCTTGACTATGCCCGTACCCGTCTAGCTAATGATGCCAAGGCTGCAAAGAAAGGAGG
TGGTGGAAGACAGTTTGATGGTCTTGTTGATGTCTACAGAAAGACACTTAAGACTGATGGTATTG
CTGGTCTGTACCGTGGATTCAACATCTCATGTGTTGGTATCATTGTCTACCGTGGTCTGTACTTT
GGACTCTATGACTCTGTGAAGCCTGTTCTCCTCACTGGTGACTTACAGGTATGTCTTGTTGTCTT
TCATTTATATCTGTAAGGTGACAGCTTAA

D

TTAACAACAACAAAAAAGGTTGTTGCATGGAAGATTTTTCACCGTTGTTTGTCTTGAAGTGAAAT
TTTAATGTCTGGCTCCACTTTTTTGTGTCAATTTTCTTCTAAGAATAAAACAAAAAGGAAAAGGA
GAAGCAATTGCATTGAAGTGGTGAACAAAATTAATTTCTCAACATCAAAGTTGATGACTTCATAC
ATATAATTTCACACCTAAGAGACTAATTTGACACTGTTAGCAAAAATAAAAATCAAACCTTCATC
ATGGGTCAACTCTTAATTAAAAATCTATCTAGATATTTATATGTAGTGTTGTTGTTTTAAGAACT
AAAACTAAATATCAAGAAAAGAAATAAGTTTGAAACGGAGCCGAGAAAAAAACAGGGTTTACAGT
TTGATATAACACCGTATCGATGGGGTGTGAAGTATAATGTTTTGATAATTACCAATCATAAAAGC
ATTATTAAAATCGATATTTTCGGCTTTAATTTGTGTCTTGGGGCCAGGAACTTTGCAAATTAATG
AGCTATTCTAAGAGTTTGAGACCTTACCACCACTACTTTGTAACGTTTTTAACTATTTTTTATCG
TTTGCCGCTAAACAGTTTATATCGTTTTTGTGTTATCCGTCAGACCCTAAAAACTAAAATGGAAA
AATACAAGTTAACTTGTACATTACGTATGAGGAAGAGACATTATAATTTGAGCAAAAAATATGAC
AGTTTTAGGGGCACGATGCTAGAGGAAAGAGATTCAAGTAAAGGTATGTCAATTTAGGTTTAAAA
TGAGATTTGGTATAATAATTTTCTTAATTGTTTTGACACTACAAGAAATATCCACATTCTTAGCA
AGTTAGAAGCGCTGTATTTGTTTATCCACATTATTTATAATAGTTTGATTTGCTATAATAATTTT
CTTAATAATTTGAAAAAAAATTGTTATCACATAGAAACTGTAATCACTATAAATAAAAATCATGA
TCCTTTTTATCCTATCATTTAGTTATAGAAATTAAAGTTCTTAGATTCTTAAAAAAGCATAGTAT
TAGAATAA

E

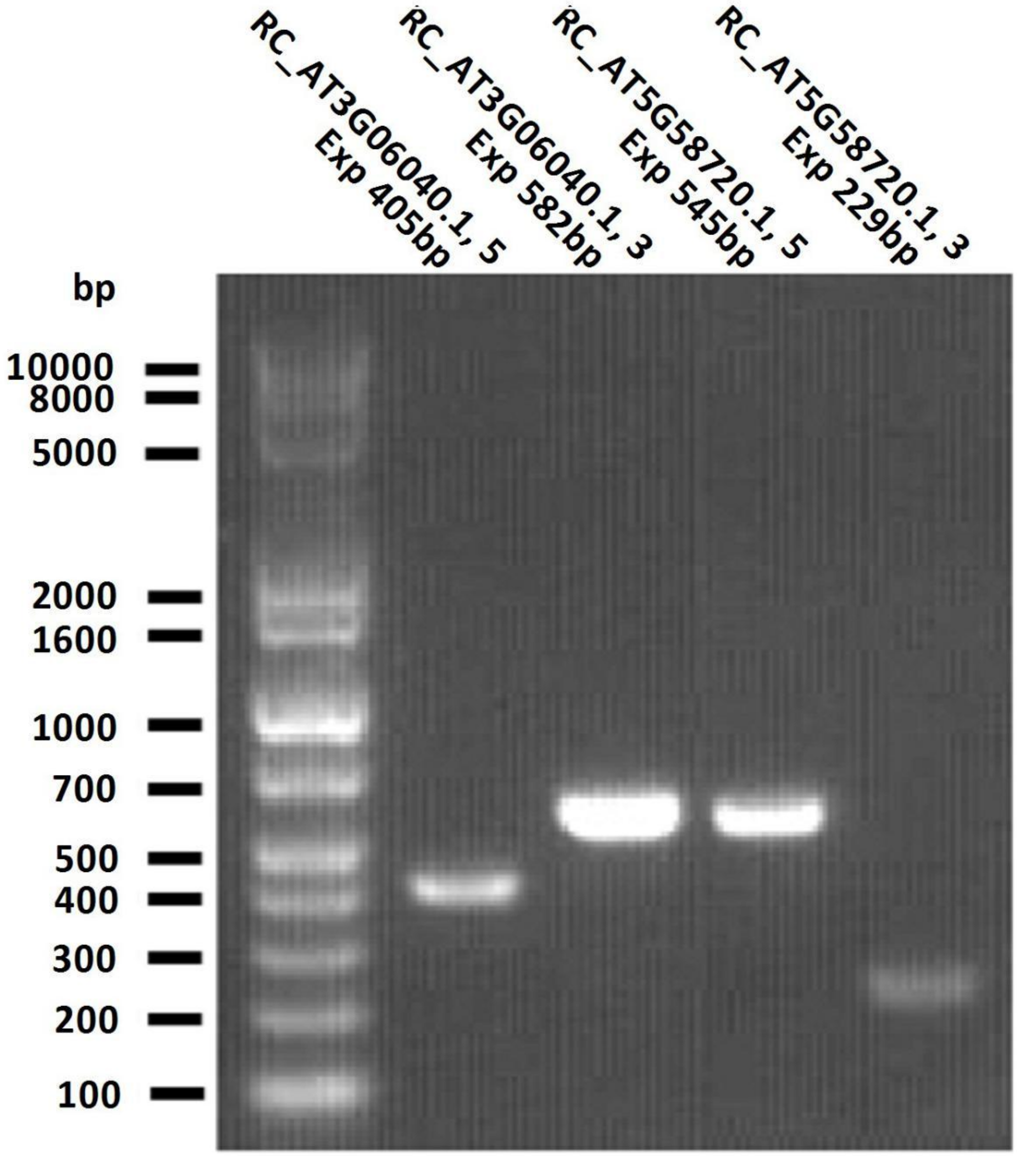
RC_AT3G06040.1, 5
Exp 405bp
RC_AT3G06040.1, 3
Exp 582bp
RC_AT5G58720.1, 5
Exp 545bp
RC_AT5G58720.1, 3
Exp 229bp
bp
10000
8000
5000
2000
1600
1000
700
500
400
300
200
100

F

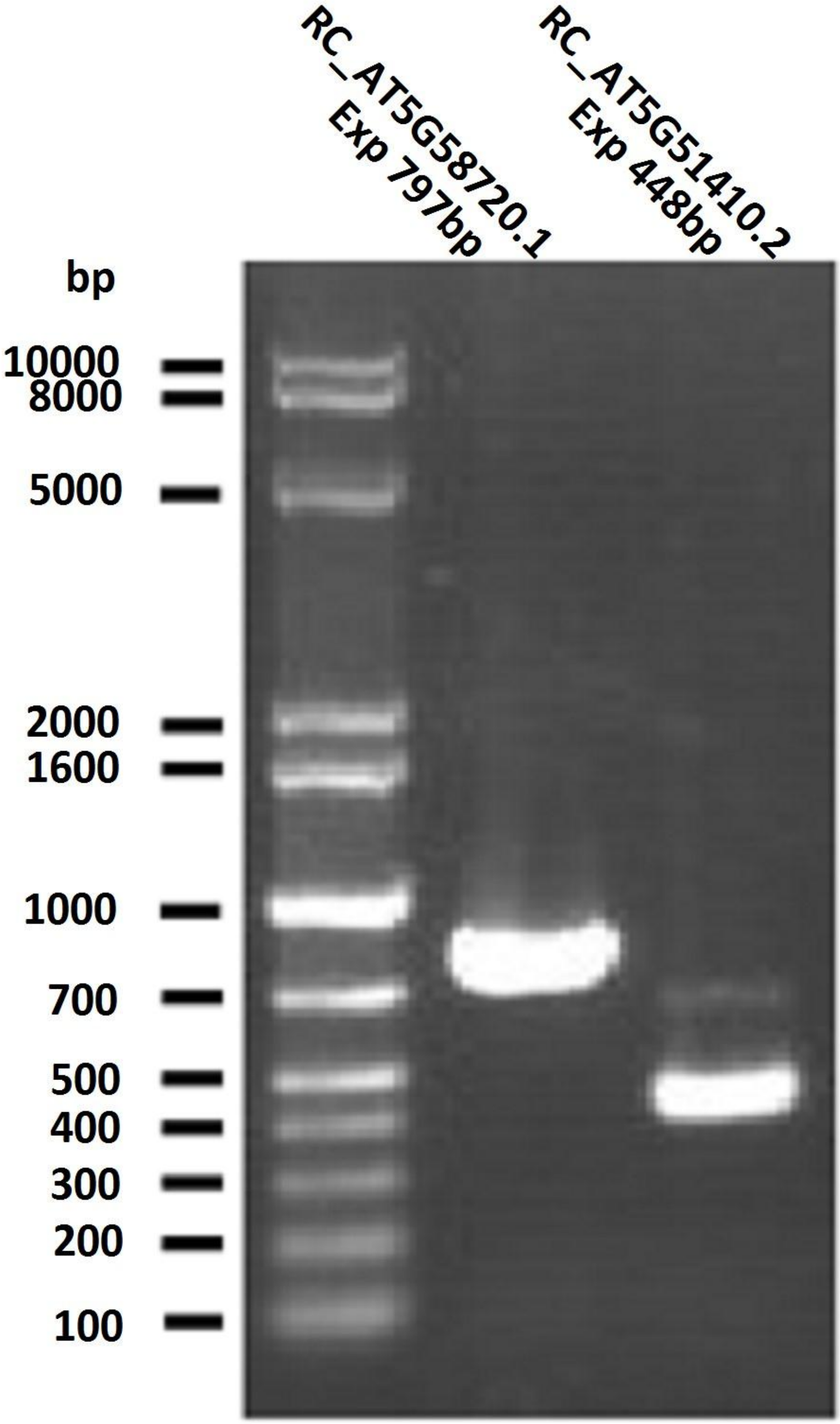

Figure S1. Assembled sequences and validation of retroCNVs in Arabidopsis.

Panels A and B show retroCNVs derived from AT3G06040.1 and AT5G58720.1, which were assembled in the accessions Can-0 and Oy-0, respectively. The underlined sequence shows the retrocopy. For each retroCNV, we designed two pairs of primers (forward primer, dark blue; reverse primer, light blue) to amplify the sequences colored in red to validate the linkage of the 5’ flanking region, retroCNV and 3’ flanking region. Similarly, Panels C and D indicate retroCNVs derived from AT3G08580.2 (No-0) and AT5G51410.2 (No-0), respectively. In these two cases, only one suitable pair of primers could be designed, which spanned from the 5’ flanking region to the 3’ flanking region. These two panels were similarly marked following A and B. Panels E and F show the PCR results performed in accessions in which four retroCNVs were initially assembled (Table 1). In E, “RC_AT3G06040.1, 5” and “RC_AT3G06040.1, 3” correspond to the amplified sequence at the 5’ end and that at the 3’ end of RC_AT3G06040.1, respectively. Similarly, “RC_AT5G58720.1, 5” and “RC_AT5G58720.1, 3” show two sides of “RC_AT5G58720.1, 3”. Finally, Panel F shows the amplified fragment derived from “RC_AT3G08580.2” and “RC_AT5G51410.2”, respectively. For both Panel E and F, “Exp” denotes the expected length of amplified segments. For all cases, the lengths of all amplified fragments are consistent with the expected length inferred according to the aforementioned sequences (Panels A-D).

A.

ACTTTGCATGTGATCGTGATAATATTTTTAATTATTATTTTTTTATTTTAGCATATGCTCCCACA
ACAATCTGTGACACTATCACACTAACATTAATATTAAAAGCACAACATGTCAATCATATATACGG
TCTTATTCGCAAGTCCTGTGATAGAACTTTTCTTCTCAGGTAGAGGAAACCAACCTTTCATTTGT
TCCTTGAAACTGAAAAGTAAAAAAACAATCGAAATTAAAGCTCGTCAGTGTTACATCGTTTAATT
AGAGCCGTTATTCGTGACTTACACTGATACCATATTAGAGTGTGGGCTTCCAACCTAAAACCAAT
TGGCAATAGGTGGAGAGGCCCATATCTTATATATACCACTTAAGATCCTACTCAACTTCCGATGT
GGGACATTGTCCCTAATACGCCCCCTCGAGATGATGGCTCTTCTAGCCATTGATCTCGATATGTT
TGGGCATGGATCGGCGGGCCAAGTATTGGGCCGGACCGATGTGGATCGGGTTGAGTATGTGCGGA
TCGGGCTCTGATACCATATTAGACTGTGGGCTTCTACACCACAGAAAAAACTTCGTCTCTCTTCT
CTGCTTCGCCCTCTCATTTCCTGTGAGATAAAGGCGGAGTCTCTCTCCAATTATTTTGCTCATCC
ATCGATTCTTA**GA**GTTCAAAATGGTTGATCAAGTTCAGCACCCCACTATTGCGCAGAAAGCTGCC
GGGCAGTTCATGCGTTCAAGTGTTTCCAAGGACGTTCAAGTGGGTTACCAGAGGCCTTCTATGTA
TCAAAGACATGCAACCTACGGAAACTACTCCAATGCTGCATTTCAATTTCCTCCCACATAGGAGA
GAAGGGGTTCACTAACTTTGCCCTTGACTTTCTGATGGGTGGTGTTTCTGCTGCCGTCTCCAAGA
CTGCTGCTGCTCCTATTGAACGTGTTAAGCTTTTGATCCAGAACCAGGATGAGATGATTAAAGCT
GGCAGGCTTTCTGAACCCTACAAGGGTATTGGTGACTGTTTCGGCAGGACGATTAAGGATGAAGG
TTTTGGTTCTCTATGGAGAGGCAACACTGCCAATGTTATCCGTTATTTCCCCACTCAGGTTTGTT
GAGTTTCATACTCTTTCTTGTTATAGCTTTTGAAAAAACATAATTTTGTGCTAACCTTCTTTTTT
GTCTATTGTAGGCCTTGAACTTTGCCTTCAAAGATTACTTCAAAAGACTTTTCAACTTTAAGAAG
GACAGAGATGGTTACTGGAAGTGGTTTGCTGGTAACTTGGCATCTGGAGGAGCAGCTGGTGCCTC
TTCCCTTCTGTTTGTGTACTCCCTTGACTATGCCCGTACCCGTCTAGCTAATGATGCCAAGGCTG
CAAAGAAAGGAGGTGGTGGAAGACAGTTTGATGGTCTTGTTGATGTCTACAGAAAGACACTTAAG
ACTGATGGTATTGCTGGTCTGTACCGTGGATTCAACATCTCATGTGTTGGTATCATTGTCTACCG
TGGTCTGTACTTTGGACTCTATGACTCTGTGAAGCCTGTTCTCCTCACTGGTGACTTACAGGTAT
GTCTTGTTGTCTTTCATTTATATCTGTAAGGTGACAGCTTAA

B.

```
FIVE            TATTAGAGTGTGGGCTTCCAACCTAA 26
THREE           TATATCTGTAAGG--TGACAGCTTAA 24
                ***:: :**.:**  *  .**.* ***
```

C.

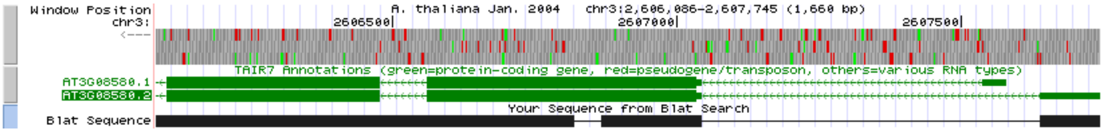

D.

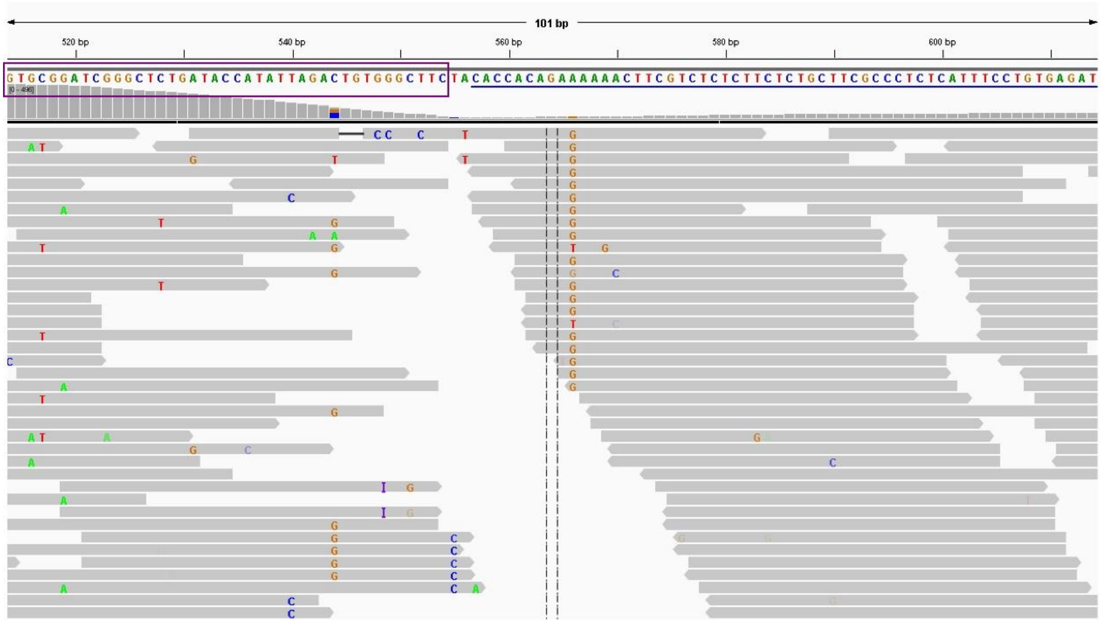
101 bp
520 bp
540 bp
560 bp
580 bp
600 bp

E.

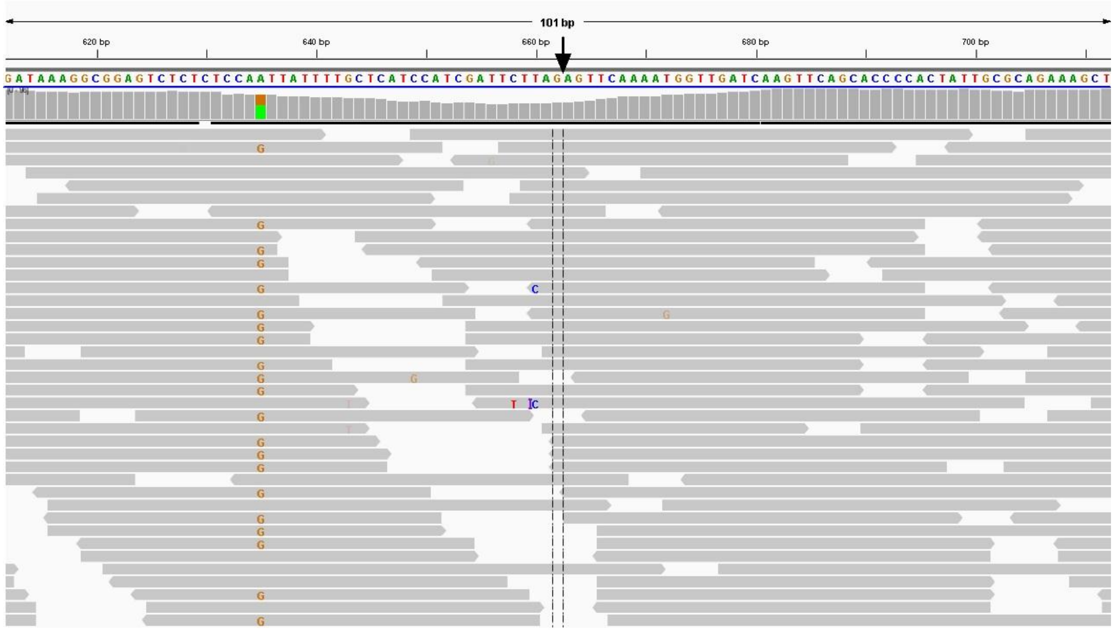
101 bp
620 bp
640 bp
660 bp
680 bp
700 bp

F.

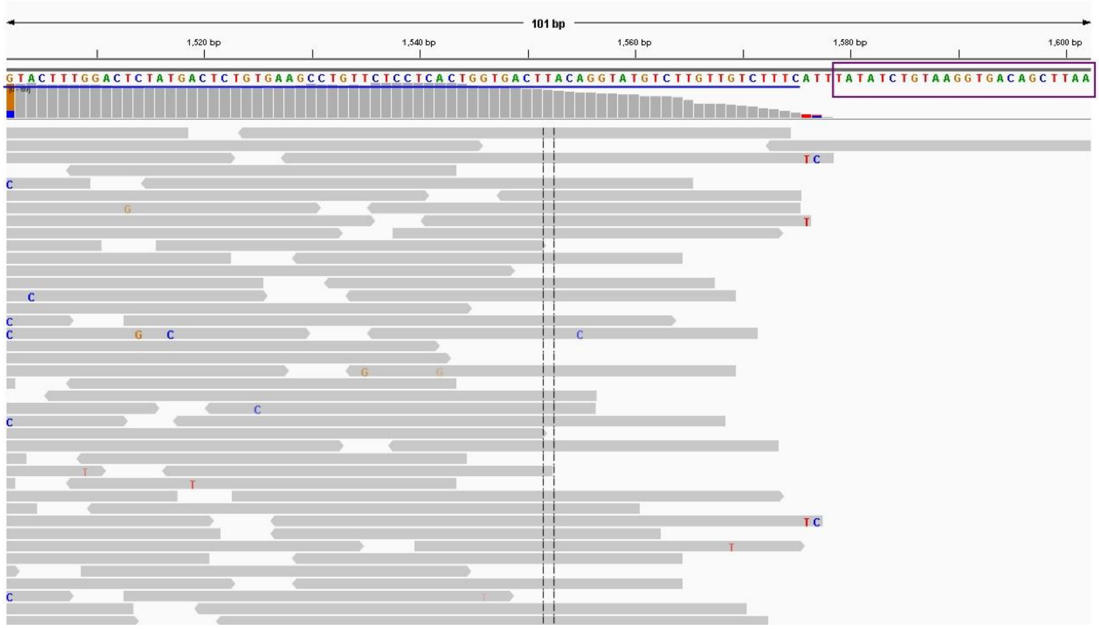

Figure S2. Schematic representation of the retroCNV “RC_AT3G08580.2”.

Panel A shows the architecture of the whole retroposed locus with the retroCNV underlined, the exon-exon junction of the parental gene in bold (“GA”), 5’ and 3' flanking LTR retrotransposon sequences in purple and the preexisting sequence in the insertion site in yellow, respectively. All other nucleotides (*e.g.,* 3’ flanking “ATT”) possibly represent secondary mutations or mutations generated during the template switch from the LTR retrotransposon to the mRNA. Panel B shows the alignment of the 5’ end of the LTR (labeled “FIVE”) and the 3’ flanking sequence in panel A (labeled “THREE”). Such a decent alignment (Identity = 53.8%) indicates that the 3’ flanking sequence is also derived from the same retrotransposon. Panel C shows a Genome Browser view (epigenomics.mcdb.ucla.edu) in which the retroCNV was aligned to the parental gene via BLAT[7]. The green boxes mark the exons of the parental gene, whereas the thin arrowed lines mark the introns. The retroCNV was represented as black boxes, with the first short line indicating a possible secondary deletion and the second long line indicating the lost intron. Interestingly, a small intron encoded by the parental gene is inherited by the retroCNV. Panels D, E and F show the snapshots in the IGV genome browser [22,23] zooming onto the 5’ breaking point, a partial region of

retroCNV encoding one exon-exon junction and the 3’ breaking point, separately. For each panel, only one 100 bp window is shown to enable a base-level view. The browser consists of the following four tracks: the axis, the consensus nucleotide with “A”, “T”, “G” and “C” color-coded, the depth curve and the reads aligned to the focal regions with single nucleotide polymorphism shown. The two black dashed lines mark the center of each view. In order to generate such a view, we prepared a customized SAM file by running BWA[24] and aligning reads onto the assembled contig in the accession “No-0”, in which the contig was originally assembled. Since hundreds of reads could be aligned to the contig, the snapshot only shows a portion of them. Consistent with Panel A, the nucleotide track is decorated with the LTR retrotransposon marked in a purple box, the retroCNV underlined by the blue line and the exon-exon junction highlighted by a downward arrow. As shown in Panel E, numerous reads span the junction directly suggesting an intron-loss event.

A.

AAAGGTAATTAAATTAAGCGAAAATATATTATCTATTTAAAAATGTATCCAAACAATTATCATAA
ATCTCAAAAATTTATATGATAACATTCCTAAATTTTCAAAGGTGTCTATCAATCTATAAAACCAT
GATTACTTTCTGAAACCTTCCAGAAATTTACTAAAAACATGTTGTTCCTAGATACTTTTGAGTTT
TGACACAATGAGGACCATTCCTATATCTTTTGTAAGATACTCTAGTAGTCTAGTCCTACTGTCTC
GATCTTCTATTAAATCTTTAAGTATAGAAGATCCAAGTAAACTTACATATAGTTAATAGAAGATC
CAAGTAAGATCAAAGCCAATAATATTAAATTTATATAAAAAAAAATTGTAAACACTGTTTTTTTT
TTCAATTGATGAAAGGAAAAAAGCATTCAGGAATTGTTTTTATTTTTATTTTTTTGTTAACATTC
ATTTATTTAAATTGCATAAGAAAAGAAAAAAGTGATAACCATTGCCAGAAACAAAGGTATAGAGA
ATGTGGTAACCATTGATCTGCATGGTCAGCATGTTAAACCAGCAATGAAGCTACTGAAGCTACAT
CTGTTATTTGGATCATATGTT**CC**AGCCATTCAGACTCTACGAGTGATCACAGGATGTGGAGCTTC
TGGGTTTGGGAAGTCTAAGGTGAAACAATC**AG**TGGTAAAGCTGCTAGAAAGAGAAGGAGTTAGGT
ATTGTGAAGAGAACAGAGGGACACTGCTGATCAAGCTTGACGGAGGTAGTAGAGAGTTCAGTTTC
TTAGACACAGAGAGTGACTCTGATGAATAAGTGATAACTAAAACTAAAGTCAGGTTTTAGCTTTA
GATCTTAAAATTTATGTCGATTTTGCCTATATCTGATGCTAGCTCTCTGTTGTTAAGTAAATGTT
GAGCAAAAAAAAAAAAAAAGTTAACAAGCCTTAGACAAAAAATTAAGAGCCCAATAACGAAAGTTG
AACTGTAAAGAAACGAAATATAACTAGTTGTAGAATTGTATATATAGGATAGCTAGTAAAAAAGA
GTGGTTGTTCTGTAACTACAATCATTTATTTTTTTGTTGACATTCGTAATCATTGTAACATACGA
AATGGAATTTGAAAATAGTGACAGTAGCATACATGTTCTAAAGAACATTGGTAAAGTAAAAAAGA
GTGACTGAGTGTTCAGTAATTGGAACAGTAGCACACATGTTCTAGATATTTAATGTTATTTATAA
ACTTCACGATACAATAAAATTTAACATAATATATTTATAAACTTATATCGATACAATATAGTTAA
CACAAAATATTTATAAAATTATTGATATATATTTTGTTATCTTGTTAACACAATAAGTATCAGTT
AATTTATAATATATTATTACTTATATATTTGATAAACAATATCATTTGAATAAGATAAAAGATGT
CGATAACAATTTTCTTATGTTGTTAAATAATTTATTATGAGCGAAAAAGTATTTTTGTCCAACTT
ATAAAAATTGAAAATAATTATGCAAAAACTATAAATATATTTTTAGTTTTTATGTTTTTAGGTAT
ATAAATTAATATAGTCCAT

B.

```
FIVE          -GTGATAACCATTGC-- 14
THREE         GTTAACAAGCCTTAGAC 17
                * * ** * **
```

C.

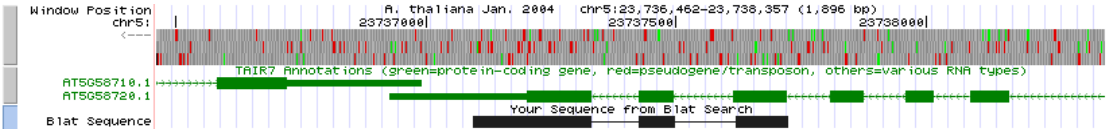

D.

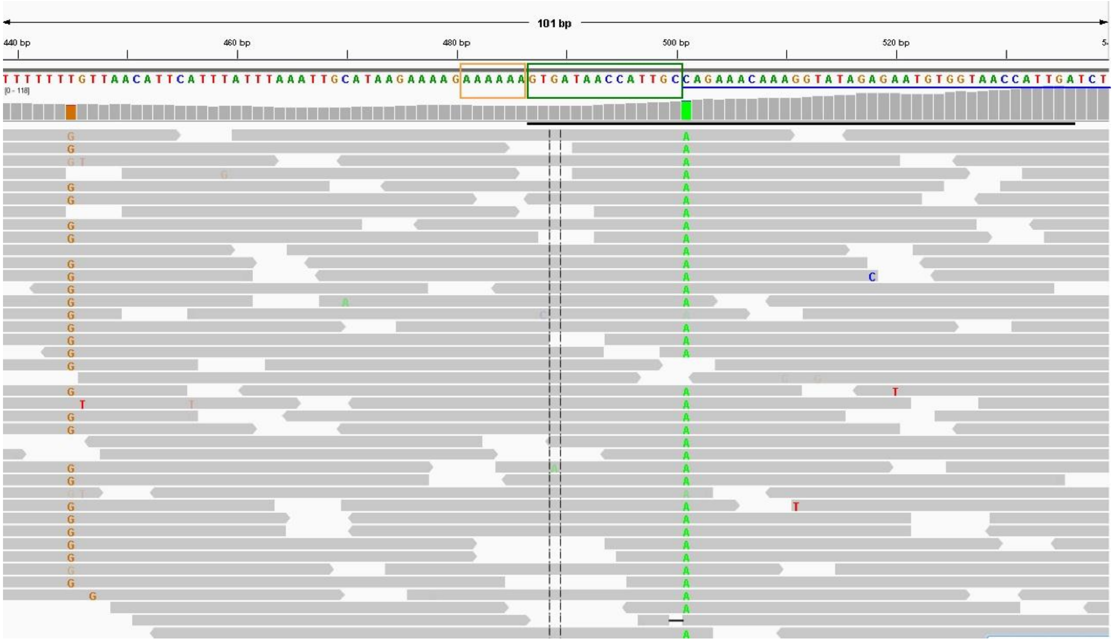

101 bp
440 bp
460 bp
480 bp
500 bp
520 bp

E.

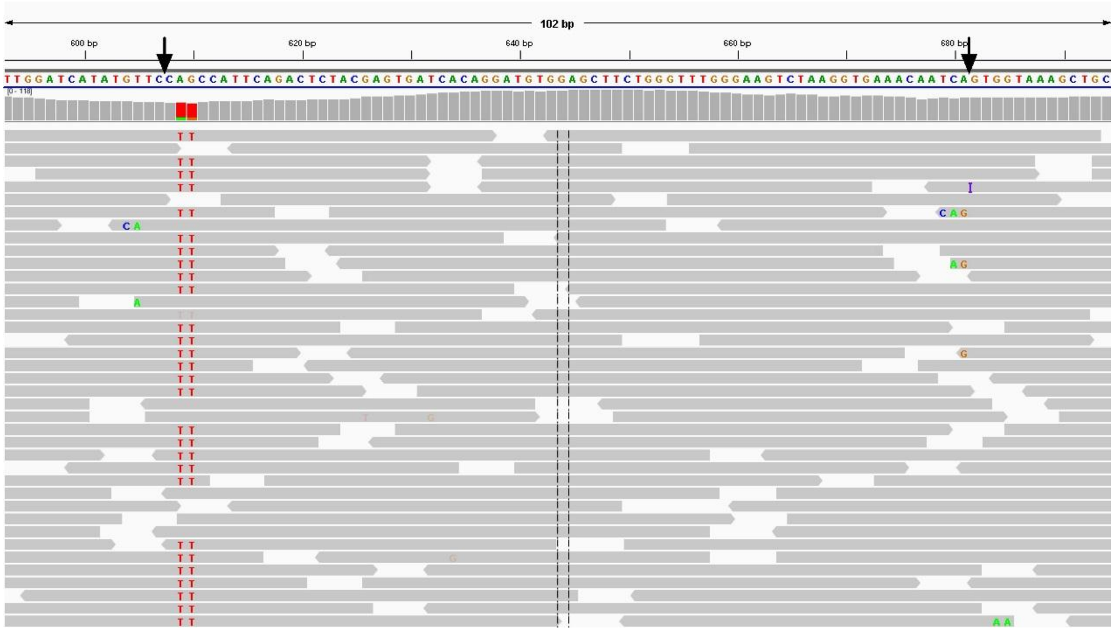

102 bp
600 bp
620 bp
640 bp
660 bp
680 bp

F.

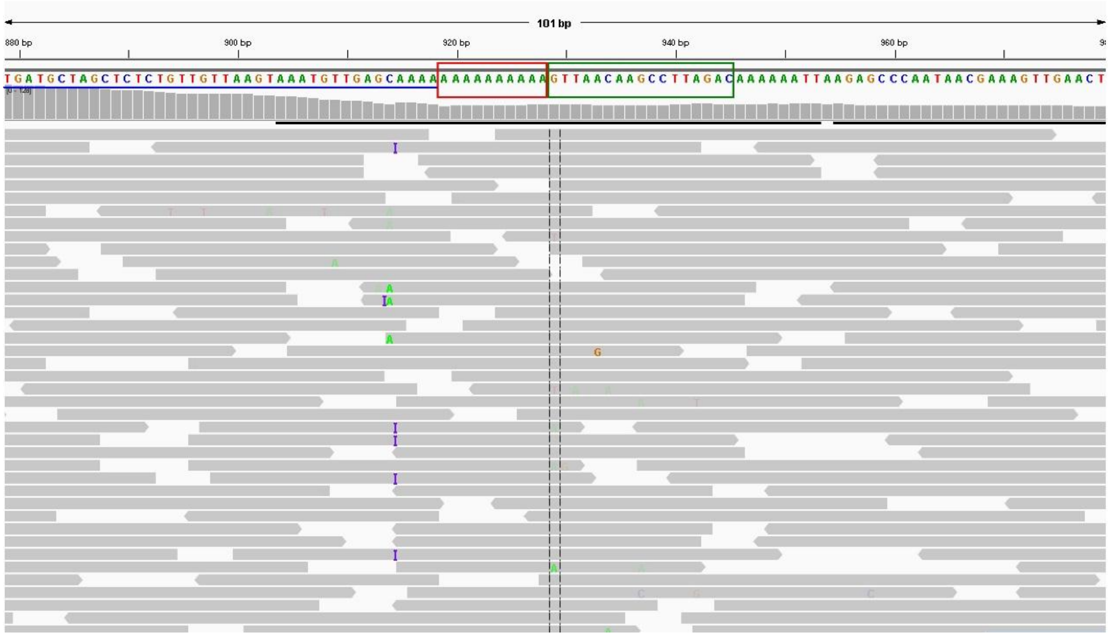

Figure S3. Schematic representation of the retroCNV "RC_AT5G58720.1".

The figure convention follows Fig. S2 except that in Panels A, D and F, the candidate target site, the target site duplication and the polyA tail are marked in orange, green and red, respectively. For Panels D, E and F, the short reads are from the accession "Oy-0" where the contig is originally assembled.

A.

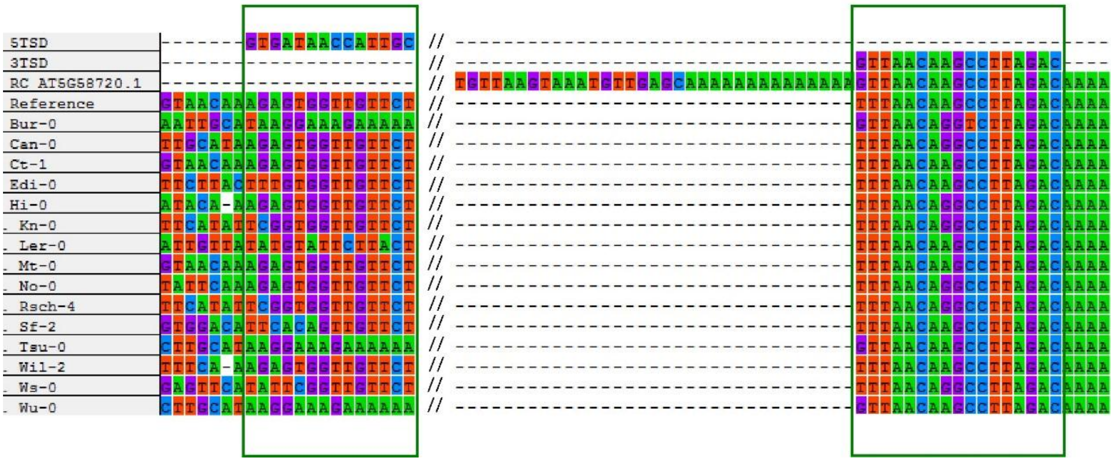

B.

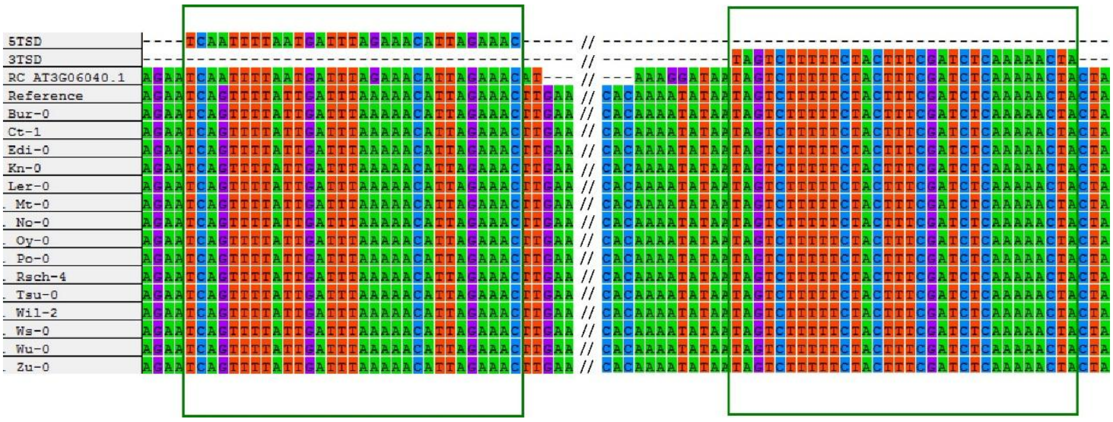

Figure S4. Multiple sequence alignment at the insertion site of the retroCNV RC_AT5G58720.1 (A) and RC_AT3G06040.1 (B), respectively. The snapshot is made by Mega[25,26] and the alignment is done using MUSCLE[27]. TSD is marked in a green frame. In A, the third sequence is the assembled retroCNV and its 3' flanking region. The last 16 sequences show the empty site in the accessions without "RC_AT5G58720.1". "//" denotes the skipped retroCNV sequence. The left side is poorly aligned compared to the right side suggesting the absence of 5' TSD. Panel B is similarly plotted as Panel A. Compared to Panel A (RC_AT5G58720.1), the empty site of RC_AT3G06040.1 clearly encodes 5' TSD, polyA tail and 3' TSD. "//" denotes the 6 kb insertion across the reference genome and 16 accessions.

A.

TCCGTTATCTCCGCCGAACCATCCATCCTCCGCTACTTCATCTCCGCCGCTGAGATCGGAATCAC
TATCCCACTCCATTTGATTTTGCATACACACCCACAAAATAAAGCTTAAGACTGCCCACGAATCT
TCTTCCTCAGCGACAGGAGAAGAGACCCAGAAGAACACAGTTTGATTTTGAATCGCGGAATCTGA
TATCTGTAGGTAATCGAAGTCTCCACAGGAAAAAGTTCAAAACTTTAGACAAACCCAGAAATCGT
CTTCTTCAAGAACAGGAGACAGAAGAACAGATTTTGAAATGGAATCAAGCGAAGGAAAGAGGAAT
CTGATATTTGTAGGTAATGGAAGTCTCCACCGCCCACTTCTAAGAGGAGGAGAAGAAGAAGAGAG
ATGATTCGGTCGCTCGATGACTCGGCTCTTCTCAGTGCCTCTCCTTTTCCATCTTTAACCAGACC
GGTTTCTTAATTTTACCTTACCGGTTTAGTTTATTTATCCGGTTTACACAAAATATCCTGTAAGT
TCCTTGTGAGATTTTTTTGGTTTTACCAAATGAGTGTATTAGAATCAATTTTAATGATTTAGAAA
CATTAGAAACATCGTGACTCAGCTCCGTCTCTCACTGTAATAATCAAGTCAGAGCCGACGAAGTT
GACGTTTGCGCCGTCG**GA**GGAGAGTTTTTACTGTTGCTGTCCAGTTGACATTTTGAGACATGAAA
TGATCTGGGGTTGATGTTTGTATGGTAACAGAGGAATTATAGTCATGAAGCTTATTTCACTTGTC
AGAAACGTTCGTTCTCGCCAATGTCAACCGGAAGTTATCTGGTCTTTGCAAGTTCGTTTCTTGCA
GCAAGATTCTGTCTCGAAAGCTAAACCCAAGAAATACAAACACCCGTCAGTTTATGATCCGTATG
GTCCTAGACCCCAGCCTTCAAGCAAAATCATGGAGCTAGCTGAGCGTATAGCTGCATTATCTCCA
GAAGAAAGAAAACAGATTGGTCCTGCTCTCAATGAACACCTGAGGCTTCCAAAACAACAGATGAT
TTCATCGGACGGCATTGGAGCAAACAAGATACGGAGCTGGGAATGTAGAGGAGAAGAAGGAGAAG
ACGGCTTTCGATGTGAAGTTGGAGAAGTTTAATGCATCTGATAAGATCAAAGTGATAAAAGAAGT
TAGAACGTTCACAAGTTTGGGTCTGAAGGAAGCGAAAGAGCTTGTGGAGAAAGGATAATAGTCTT
TTTCTACTTTCGATCTCAAAAACTACTAAAAAGTGAAGCCTTGTAAATCTTCTTTAAAGAGTAGA
AACATGTATTATTATCTTTTAGTTATTCATCTTCAAAGTTTTGATTAATTTAAAGTGAATGAAAT
ATAAATTCAATACAAAAAAAGAAGAAACCCAAATCTACAAAAGAAAAAAAGAAAAATATATAATT
GATTTAGGATTCTAAATTCTCACGTACTCGGAGAGCAAGCCGTTGAATAGACTGATCATCGAAGC
AAGGATCAAAGGTCTTAATCTTGGACACAAAATCCATAGCTTCACTATACTTCCCTGCTCTGCAA
TAACCATCTACTACCATTTTAAAAGTCAGCTCATTTGGTCTGCAATCATTCTTCGCCATGCACTC
AATCACATCTTCTATCTCTGCAAACATTCCCATCGCCGTGTAACCCGAAACAAACGTGTTGTAAG
TGAAAATGCACGGTCTGATTCCCCGCTCTGTCATCTCAGACAGCATCCTAACCGCTTCTTGCATC
AACCCTCTTCTGCAGAAACCTTTGATCACTGTGTTGTAAGAGACCAGGTCCGGTTTTAACTGCGA
TTTTTCTAGAGTCTTGAGGATTTCTTCGGCTTTCCAACACTCTCCTCTTCTTACGTACATGTCCA
TCAGGCTGTTGTAGGTTACAAGATCCGGGCTTAGCCCATCCTCGCGGATAGACTCGAGAATCCCC
TCTGCTTGATCGTACATGTTGTTCCTCGTGAAAATGGAGAGCATTGAGTTGAAAATCACCATGTC
GGGTTTGTATCCGTGCTTTTTAAACAATGTGAATGCTCTCTCTGATCCTGCTAGTGCTCGGCATT
TAAAGTTTGCGAGGAGTAATGTTCTCAAAAGCATCCAGCTCGGGAATATTTGGCCCTCCTTTATC
CCATTCTCGATTCTCTCTATCCCTAGATAGTTCCCTCCTTTAGCATAACACTGAAGCATCAAGGA
ATAAGATGTTTCAGTAGGTTTGAAACCTTTACTTTTCATGTCGGAAATCACATTTTCGCCTGATC
TCCAATCTCCTTTTCTTGCCAAGGCATTAAGTAACGCGTTGTAAGTCGTAACGCAAGCGTTGAAC
CCTGCTCTTGTCATCTCACCATACATTTTCGATGCATCAACCTCTGAACCACATCGCCCATAGGC
ACTGATCAGCGTGTTGAATGTGTCCCTATCAGGTTCAAATCCGCAGCTCTTCATTTCACGGAACA
CCCGGTTCACAAACTTATCCATACCCTTATTCCCACACAGAGCAAGCATTGTGTTCCAAGTGGCA
CGATTAGGGG

B.

```
FIVE            TGAGTGTATTAGATCAATTTTAATGATTTAGAAACATTAGAAACAT 46
THREE           T-AGTCTTTT---TCTACTTT--CGATCTCAAAAACTACTAAAAAG 40
                * *** *:**   **:* ***   *** *..***..*:. ***.*
```

C.

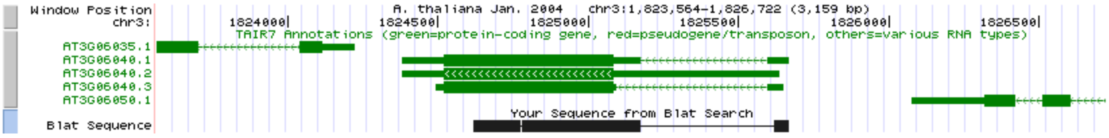

D.

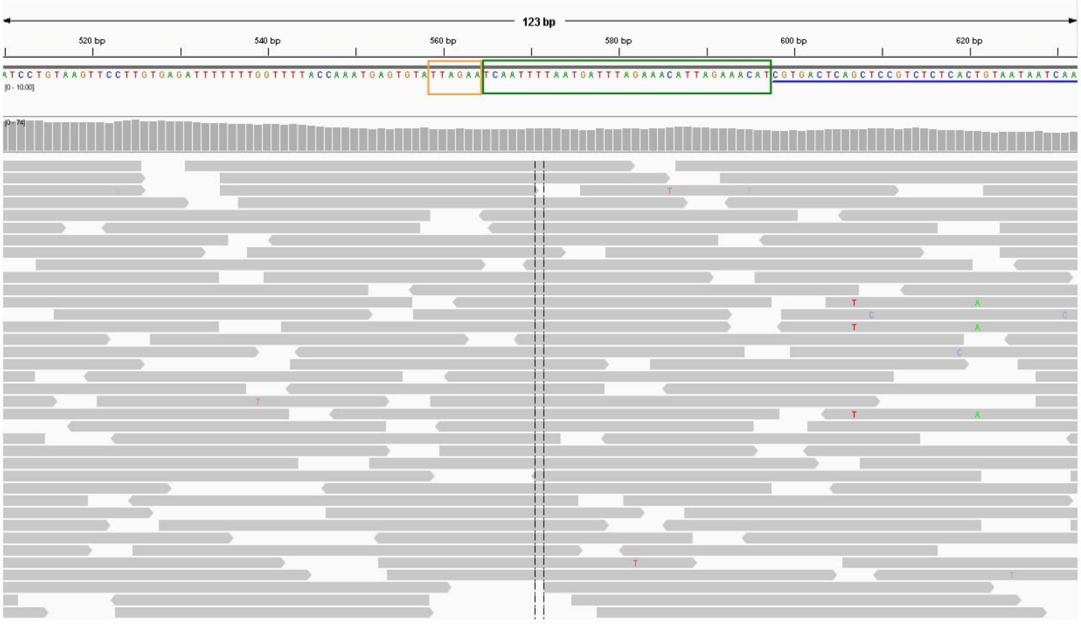

E.

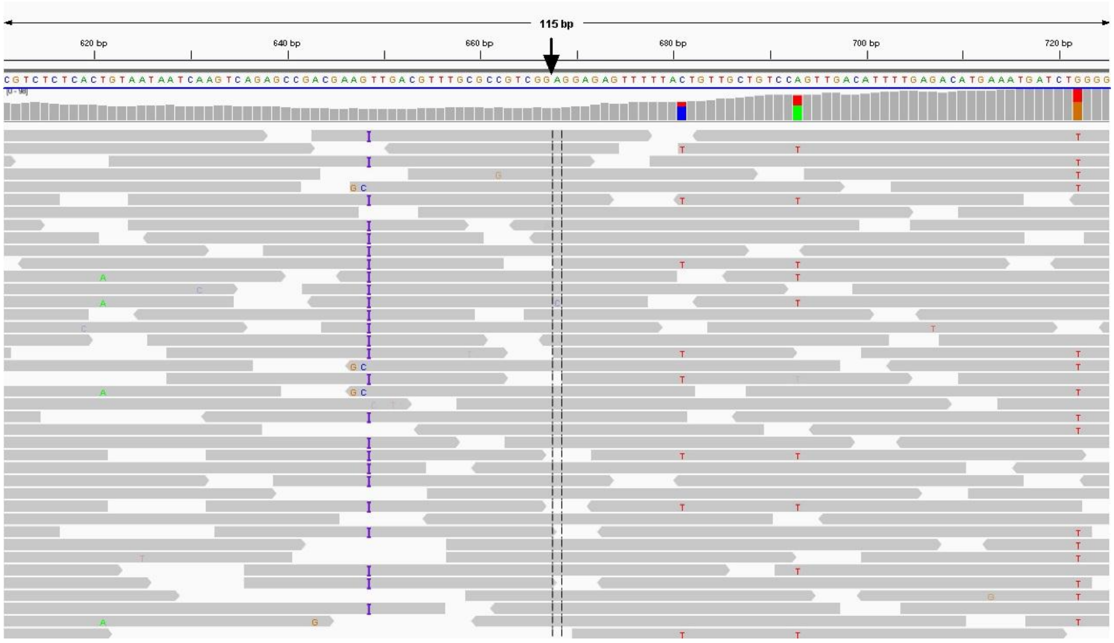

F.

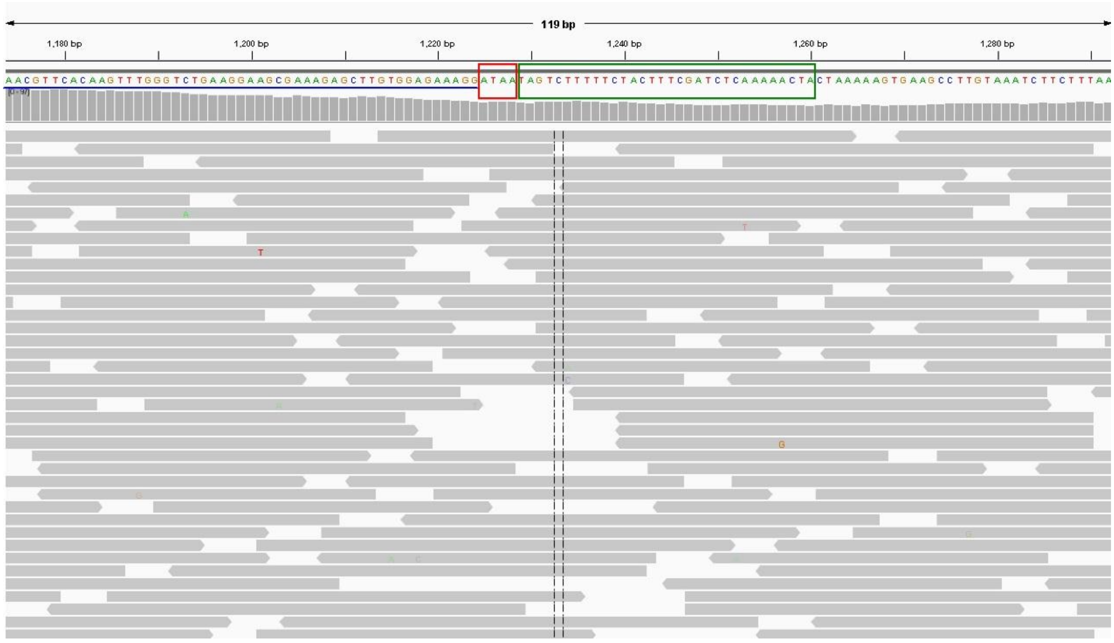

Figure S5. Schematic representation of the retroCNV “RC_AT3G06040.1”.

The figure convention follows Fig. S3. The short reads in Panels D, E and F are from the accession “Can-0”.

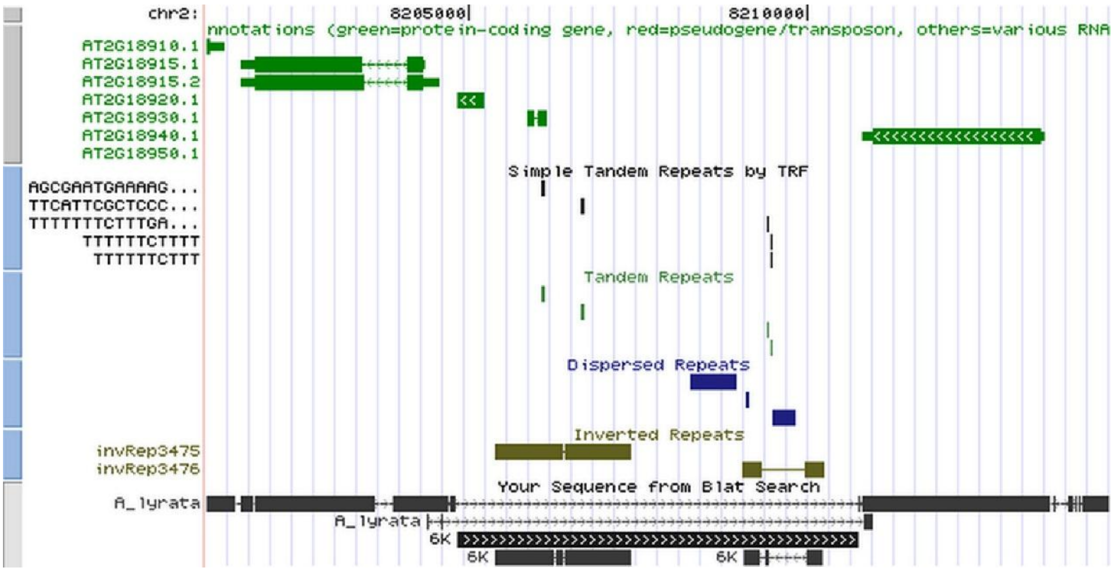

Figure S6. Genome Browser view (http://epigenomics.mcdb.ucla.edu) of the alignment of *Arabidopsis thaliana* and *Arabidopsis lyrata* in a 13 kb region around the insertion site of RC_AT3G06040.1. The following tracks are shown including gene annotation, various repeats and user-supplied sequences aligned to this region. Sequences tagged by "A_lyrata" refer to the orthologous genome sequence of *Arabidopsis lyrata* identified via BLAT[7] search using the sequence flanking the insertion site of the retroCNV at the reference genome, while '6K' is the insertion between two "TSDs" of "RC_AT3G06040.1" in the reference genome. Clearly, this insertion is absent in the *Arabidopsis lyrata* genome.

A.

TTAACAACAACAAAAAAGGTTGTTGCATGGAAGATTTTTCACCGTTGTTTGTCTTGAAGTGAAAT
TTTAATGTCTGGCTCCACTTTTTTGTGTCAATTTTCTTCTAAGAATAAAACAAAAAGGAAAAGGA
GAAGCAATTGCATTGAAGTGGTGAACAAAATTAATTTCTCAACATCAAAGTTGATGACTTCATAC
ATATAATTTCACACCTAAGAGACTAATTTGACACTGTTAGCAAAAATAAAAATCAAACCTTCATC
ATGGGTCAACTCTTAATTAAAAATCTATCTAGATATTTATATGTAGTGTTGTTGTTTTAAGAACT
AAAACTAAATATCAAGAAAAGAAATAAGTTTGAAACGGAGCCGAGAAAAAAACAGGGTTTACAGT
TTGATATAACACCGTATCGATGGGGTGTGAAGTATAATGTTTTGATAATTACCAATCATAAAAGC
ATTATTAAAATCGATATTTTCGGCTTTAATTTG<u>TGTCTTGGGGCCAGGAACTTTGCAAATTAATG</u>
<u>A**GC**TATTCTAAGAGTTTGAGACCTTACC</u>ACCACTACTTTGTAACGTTTTTAACTATTTTTTATCG
TTTGCCGCTAAACAGTTTATATCGTTTTTGTGTTATCCGTCAGACCCTAAAAACTAAAATGGAAA
AATACAAGTTAACTTGTACATTACGTATGAGGAAGAGACATTATAATTTGAGCAAAAAATATGAC
AGTTTTAGGGGCACGATGCTAGAGGAAAGAGATTCAAGTAAAGGTATGTCAATTTAGGTTTAAAA
TGAGATTTGGTATAATAATTTTCTTAATTGTTTTGACACTACAAGAAATATCCACATTCTTAGCA
AGTTAGAAGCGCTGTATTTGTTTATCCACATTATTTATAATAGTTTGATTTGCTATAATAATTTT
CTTAATAATTTGAAAAAAAATTGTTATCACATAGAAACTGTAATCACTATAAATAAAAATCATGA
TCCTTTTTATCCTATCATTTAGTTATAGAAATTAAAGTTCTTAGATTCTTAAAAAAGCATAGTAT
TAGAATAA

B.

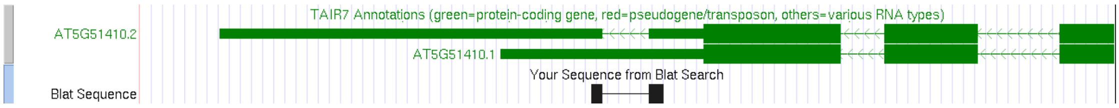

C.

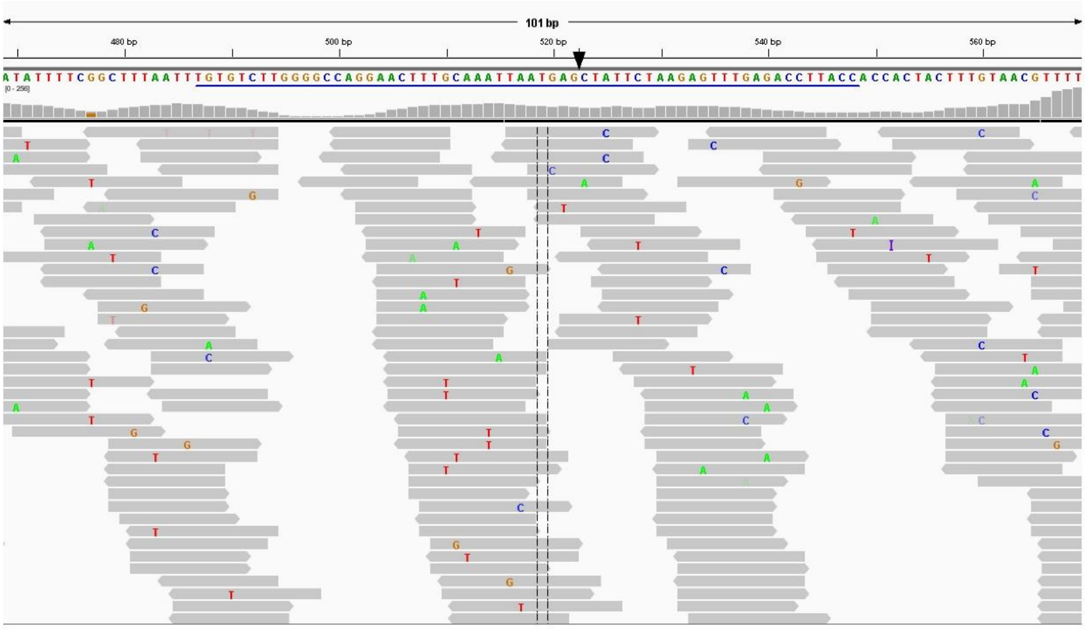

D.

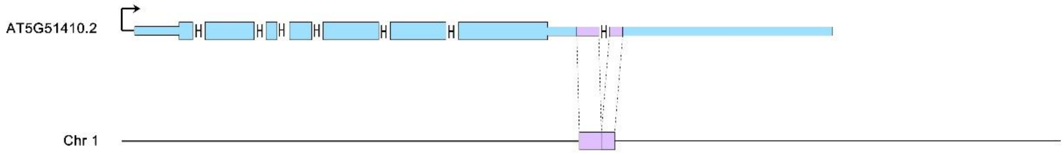

Figure S7. Schematic representation of the retroCNV “RC_AT5G51410.2”.

The conventions of Panels A, B and C follow Fig. S3. Panel D follows Fig. 1. The short reads in C are from the accession “No-0”.

A.

ATAATTTTGCTTAAACATATTTTTTGTTTTGCTTATTTGATGGACATAAATGTACAACATCCTAT
TGGATTTGAATCTCATGGAGATATTGCAACCCTCTTCTCATGGTGCTGGGATTTAGCGGGACTGA
AAGGAATATCTTTTTACGTACGTTTAAGA**GG**TATGGAACTGGAAACTTTGATTGGAAGGAGTTTG
TTAATCCCTTATACATGAAGACCTATGACGAGATAAATA**AG**TATGGAATATTACTTTTGAAACAC
ATTGCCGAAAACCCTAACGATAATCCTAAAACTTTTAAA**GA**TGGAGTTCCCAAAGAAGGGATAAG
AAGTGACGAGCTACTAGTGAGCATGACTTTTATGATGCTAGTAAAGGAGAA**GT**GTCAATTTTTGG
ACAACAATCCGACCAAACCTGTTTTCTCTGACTACTTTATCAGAAAGTACAATTTGAGAAGTGAA
GCATTTTCTAAGGAAGAACATGATAGGATGCTGATTCTTGCTGTTTCCA**AG**CATGGCTATGGGAG
ATGGGTGGCCATCGTTGAAGACGAAGAGTACAAAGGGCCTTGGCTATCACATCAGATTAGAGGTG
CTCGGGTACGAAGATCCTTAACAAGAAAACCAAACGGATCAAATTCAACAGAGCAGAGATTATTA
GTGGTAAATTGACGAACAAAAATTAGATTTTTTAGAATTTGAGGACAAATAAGAACATTATTGAG
GTGAAGGGGACGAGAACTAGATGGAATTGTTGTTTGACCATAAGAAGTGACAGGAATTGTGGAAC
CGTTACCGACAGTGACGAGAGGAAGAGAAGAATTAGAGGATGAGGTGATGGAAGAGAGTGTACCT
GGCTGAGCCATGAGGTGAGCTGTAGCTGCGGAGTCCATGTACCATCCAACATCATTAGGATCCGA
GAAAGTCATCGTGTTGAATGCATTTGCGAGCGTAGTGGGAATTATGTCTGTGTTTGCTGGAGAGG
TGATTGGTTGGGCCGAGGTGAGGAAGGCAGACCCGTTTGATGGAGGTGCGTGACCGAGAATCCCT
TGTGCGCGTTGTGGCAGCGTTGGTGGTTGAGAGCCAATGTGAAATCTTGGTTGCCCATAGAATTG
TGGAGGAGGTGGTGTAGTGGGCCAAATTGGCATATTGGGCCATTGTGGATAACTTGGCCATTGAG
GTTGGTTGGGCCAAGTTGATGTTTGATTTCCAGAGCTCCAATTACCGCCGTTGTAGTTAGGGTTG
CCGCCACCACCACGACCTCTGTTTCCTCGACCTCCTCCGCGATTGTTTCGATTGTTTCGTCCTCC
GCCACGACCGTACTGGAATTGTTGGTTTGTGAGGACTGAGCGATAGTCTTGTTGTTGAGAAGAGG
CAAGAAGAACATGTGGTGAAGAAGCGTTGTCATCATTGTTAGGAAGAGATTGCAGCTTGGTTTTA
AGCCTCGATTCTTCTTCAATGAGCATCGATCTGGCATCGCCAAAAGAGCAAGGTGGGGAACGATG
TTTAATAACATTAATGATGTTATCAAATTTGGAGCTAAGTCCATTGAGAAGGTGCATCACTAAGG
CGCGATCTGAGACAGGGGAATCCACATTGGCGAGCGTATCAGAGAGTGACTTCAGTTTTTGACAA
TAGTCATGAACGGATTGATCGCCAATCCAGAGATTTCGGAGTTCATTTTTCAGTTGTATTGCACG
GGCTTCTTTGTTGTCGAGGAATAAATTCTCGAGCATGAGCCAAAGTTCGCGTGCAGAGCACTGCG
ATTTTAAGACAGAGTTGAGGAGCGATTCTGAAATGGTACCGTAGATCCACATCTTAACCGTATTG
TCAAGTTGCTTCCATGTTGCGTCGGTTGGTCCGATGGGAAGGGAGGTGCCATCGATATGCCCGGT
GAGAGAGAAACTAAGGCAATGAGTTTCGAAGAGGATGCGCCATGAATCGTAGTTCATCTTCTCCA
TGTTGAGAGTGATGGGGATGTGAGCACGGATCTGAGTGAGACTCGCAGCAGGAGTAGTAGCGGTT
GGTGGAGGAACTGTCGCCATGAATTAAGTGCAGATCAAGAGAGACAAAGAAAAAAAAATTGGAGA
GAAGAGCTAGAGATACAGAGAGAAAAGTGAAGCGAAGAGCAAAAGAGAGAGAGAGAACACGAAGA
AGAAGACTGAAAAGATGAGGAAAAGATGACTGCTAGAGTTTAGAGAAGAGTCTCTGATACCATAT
TAGAATAGGTATAATCTCAATTGATGAGTTCCTTGACATATTCACAAAGGGTTTAAATACATAGC
AATATACAAAGTAGCCGTTAGAGGCTTAATGAGAAGATTCCTAAAATACAGAAAGGAATATATGC
ATTCCTATTCTAATAATACTTTTCCAAGAGGTTGCCTGCAAAGACCTGAATATCCATTTCCCTTC
TGATACTGAGTCTGCTCATAAAAGAATTCGTGATCATGTGGAAAAACGGGTTAAGAAGATGGAAG
ATGCGATAAAGTATGAGTACGC**AG**AAAAGATACGTGCTGAACAAGTATAAGCTGAAACAAAGGGA
ACGAGCTTTGTTGATGCAGACAAAGAAATGCTTGATAGACTGCCTAAGAATGATCCCATC**AC**TTC
AGAAGAAATTTCTGAAGCTGCTGTTGACAACAAGCAAAGTAGAGTTATTATAGACATATTTAGAT
ATTCAACCATACGATCA**GA**GTGTTAATAAGAAGTCATTATAGACATATTTAGATATTCAACCGCT

GATTCGAATGCCGAGGAAGAACTTTAGGCCTCTGAAACCTATCAGTAAGGAAATAAACACTAGGC
TAAGCGCCGCAACAGATCATGATGTGGAGATAGATGTAGCGGATAACATCATTGTGTTGAATTGA
CATCAAAATCATTATTACTTCTTTCATTACAGTTTTATCTTTGGCTGATTTTATTTACTAAGATA
CCTTTCGCTGAATACTTTTTTTTTGTCAAAATTATAATAACTAAGTTACCTTTTACTAAAAAAAAG
GAAAAAAAAAAAACGATATTGCAGCCCTCGTGCCGTCTTATGTACTGTAGCCGTACGTTTTTGTC
AAGTTCGATTCCTTACACTTAACAGAATC

B.

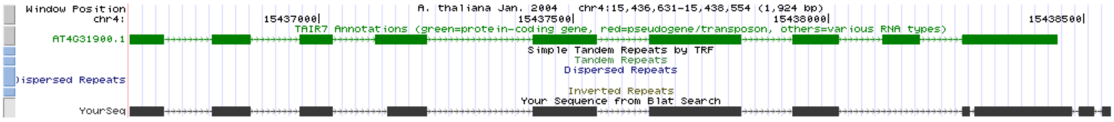

Figure S8. Schematic representation of the retrocopy “R_AT4G31900.1” (“R” is short for recently evolved retrocopy) encoded by the Arabidopsis reference genome.

The figure convention follows Fig. S3 except that 1) in panel A, an insertion is marked in dark blue which can be aligned to LTR element *Copia-82_ALY-I* on Repbase[9,10] with the BLAST *E* of 5×10^{-49}; and 2) in panel B, the retrocopy was encoded by the reference genome itself, and it was further aligned to the parental gene, *i.e.*, AT4G31900.1.

CAGACGCGGACGGGGACTACGAGTCTGTATCCAGGAAGCGTGCGAAGAAGACGACGAGTGCTAGT
ACAGATCTTAAGTTGGTTTCTATAGACTACTTGCGGATTTCAAATCTTTTAAAATATATTTTCCT
TATTTTAGTATTGCTTTTAATCCTTGTTGTACTTATGTCTATGCGACTTTTTCTTGCTTGAAAGC
ATGCGGTCTTTGTTTTATGCATCAAATTCGCCTGTCCCCCATTAGCATTTTTTTCTCCAAAAAGG
ACCAGCACTTATGAAACATATGTAGGTTTTTGGAGAAGACTTCAAGAAGTGGTTTGTGTTCAATC
AAATCAAATATTTCCAATTCTAGAAGTCTTAAGAGTTTACTTTTCATAAAAGGAAGAAACATGAA
GACGAACAAAAATTTCCTATTCCAAATAGCCTCAACCTATTAAGTTTAAAATAGGTGCTAAGGCT
AGAGAAAAATTTAGTTTGAGTGAAACTTGTAAGTGCACTAGGGTTCCCTACTTCGTCCAGCGAGA
AAGGAAGAGGGGTTTCGTCTGATTCCGCTAAATCTCTATCTTTATTTAAAGATGATGGATTCCGA
TGATGATATGCTCGATGCCCACGATATGGACTCGGTAGATTATGATTTTGACAGCGGCGGCACCG
ATGATGACAACGATATTGATGAAACTGATTACGTGTTTGGTGAGGCTGACACGGACGATGCCGCC
ATCATCGCCTACCATCGCTC**TC**AGATAAATTATGTTGTTCTCAAGGAAGAAGATATTCGCAGGCA
TCAGAAGGATGACGTTGGGCGAGTTTCCGTTGTCCTCTCCATAACCGATGTCCAAGCAAGTCTTT
TGCTTCTTCACTATCACT**GG**AGTGTCAGTAAAGTTAATGATGAATGGTTTGCGGATGAGGACAGA
GTTCGTAGAACTGTTGGCATATTAGAGGGACCTGCACCTGATGGCAGA**GA**GTTTACATGTGGAAT
ATGCTTTGAATCCTACCCTCTTGAGGAAACTATATCGGTTTCTTGTGGTCACCCATTCTGCGCTA
CATGTTGGA**CG**GGTTATATAAGCACAAGCATCAATGATGGCCCAGGATGTTTGATGCTAAAATGT
CCCTACCCTTGTTGTCCTGCGGCCATTGGTCGAGATATGATCGATAACTTGTGTTCCAAGGAAGA
CAAGTAGAGGTATTATAGATATTTTCTTAGGTCTTATGTTGAAGTCAACAGAGA**GA**TGAAGTGCT
GTCCTGCCCCAGGATGTGAGCATGCAATTAGTTTTGCTGCTGGGACCGAAAGTAATTATGATGTT
TCGTGCTTGTGTTCGCATAGCTTTTGCTGGAA**TT**GCAGTGAAGAGGCTCACCGTCCTGTGGATTG
TGACACAGTTGGAAAATGGATACTAAAGAACAGCACTGAATCTGAAAATATGAATT**GG**ATACTTG
CCAATTCGAAGCCTTGTCCAAAGTGTAAGAGGCCAATAGAAAAGAATCATGGATGTATGCACATG
ACATGCACACCACCTTGTAAGTTTGAGTTTTGTT**GG**CTCTGCCTTAACGCATGGACAGAACACGG
GGAAAGTAGTGGTGGGTATTATGCCTGCAACCGGTATGAGGCGGCTAAGAAACAAGGGTT**GT**ATG
ATGAGGCTGAAAGGAGGCGAGAGATGGCAAAAAACTCGCTAGAGAAATACACTCATTACTATAAA
CGATGGGCAAGCAATCAAGT**GT**CGAGGCAAAAAGCTATGGGGGATCTGCAGAAAATGCAATCAGA
GAA**GC**TTAGGAAGCTTAGTGACATACAGTGCACATCAGAATCTCAGCTCAAGTTTATCGCAGAGG
CTTGGCTCCA**GA**TCATTGAATGCAGACGGGTACTCAAATGGACATATGCATATGGATACTATGTA
CCAGATGATCATACTAAGAAACAATTTTTTGAGTATTTGCAA**GG**GGAGGCTGAGTCAGGTTTGGA
GAGGCTCCACGAATGCATAGAGAATGATATTGAGGTGTTTGAATTTGGTGAGGGCCCTTCAGAGG
AATTCAATCATTTCCGGACAAAATTAACTGATTTAACCA**GC**ATAACAAAAACCTTCTTCCAAAAT
CTGGTCAAAGCTCTGGAGAATGGTCTTGCTGACGTGGATTCACATGCTGCTAGCAGCAAACCAGC
AAACTGTAAACCTTCTAGCAATACAAAAGACGGTGGGAAAGGTAAAAAGGAAGCTCTAACGATGG
CGGGTTCAGCAGAAACCTAGATGGCAATTGAGATCAGCAAATTGGAGAAAGGTTTGGAGTTTAGA
ATACTTTTGAGTACACTCCTGAGAGTTTGAAGGCTATTAAAGTATACTCCTGTGAAGTTTCTTAT
CTGAAAAAAAAGAATACTAATTATGTTTACAACAATTTCTTTTATTTCTTTCAAAATTTTTGCAT
TGTAACACTTTATTTTACAGTCAAAGTTTTATGAAGTTCTATGATCTTTCTCAATGTAGACAAAG
CAATGACAGCTTCTGAGAAATAACATTGCCGTAATATATAATGCAAACGTTTATTGTAATAGTAA
AGCTCATAAGCAGAGGCAAAACAATCTGTGATCATTTTAACATATCCCACTACTAATTTCAGTAG
GTAAATAGTTCAGAGTAAAGTGTCTTTAAAAGGATTCTTAACGTGTCTCTCAAGCAGCCAAACTC
TTGGAGGCAATCTCAAAGACATTCTCAGACAGCCCATTAGCAGACATTATCATTTCCAATTGTGC
CTGCAAACACAACACACAAACACAACAGCTTAATCTAATGATTACGAATCATGGCTACAGATTCC
GATCTGCGGAAATATTGAACACAGAGGCTCAATGAAGCAAAAGAACCAGGA

Figure S9. Schematic representation of the retrocopy “R_AT1G05890.1” encoded by the Arabidopsis reference genome.

The figure convention follows Fig. S3A.

TTCAAAGGTGATGGGTTTTCAGAGGAAACGTCATCTTCATCATCCGAAGAAGAGTTTCGTGCGAT
TTCTTTACCTTCTTCGTCGCTTGAAGAAGCACTCGGCGGATCTTCTAACGGATTGAAACGTCTCG
ACATTGTTTTAGGGTTTATGGGACTTTGGAGAGACTTAAGAGGTTGAAATTGAGATTTTTGCTTT
GCTGCCTTCACCACTTCGTTCTTAGGGTTTATATATATTGAACAAAGTGTCTCTCTGTTTTCCAA
AAATAAATAATCAAAAAATTATTGGAAAACGTATAAAATTATTTATTGTTTTTCCTCTTTTTTTG
CATGACGTTTAGATTTCCGATTTCCTTCCTTAAAAATTTGGAAATTAGCTATATTGACCAATTTT
ATTTCCCTATATTTTATCTCTTACAAATAAATGTTAGATCTCTCTTTTCACAACAACACCCGAAT
ATGACCGCTCATTCAATCGTCGCACACGTTGATAGCGTCCTTCCCAAGAAATCTCGTGAGATCGA
CCGTCGCCGCCGCAGACGGAAGCGGAAGAAGAAGAACAAAGCATCTCAGGCCGATGTAGATGCAA
TGGACGTGTCAAAATCTCTGTC**AA**GCACTCCTACTGGTATTGAGACACCGGATGCAATTGAACTT
CGTAAGGAACAGAGAAAGGAACCTGATAGGGCTCTATACCA**GG**TACTTGAAGAAAAGGGAGAGAG
TGTTGTTGCTCCTGGAACATTGCTGAGAACTACACACACATACGTTATTAAGACTGGTACTCAGG
ACAAGACGGGAACCAAAAG**GG**TTGATTTGCTGAGAGGGCAAAAGACAGATAGAGTGGATTTCAGT
TTACAGCCAGAAGAGCTGGATGCTATGGGAAATGTTTTACAGTATGAGGAGGCAAGAGAAGAGGA
GAAATAGCGCAATAAGCCAGTGGACTTGAGTGACATGGTCGTCGA**GC**ATGTGTAGCAGAATAGTA
GGAAGAGGAAAATGCATGGCAAGGAAGAGAAGAAAAAGAAAGATTTCAACTTCTGAGGTGCGTTA
TGGAAGAGACAAAAAAAAAAAGATTTTAGATTCTGGACATGAGACATAGAAAGAGATTCATGTTC
CAGAATTATTAGTCTTGAGTAAGAGATCTTGAGTTTTTAGTCTTAATGCTTATTAAAAAAAAAAA
AAAATGTTAGACCACCAATGATGTAAATTTTTAAATAGGTGATTGCATCATTGTGTAAACCTAAT
ATTTACATTTTGAAGGATTTGGTCTTTCTGCAGTATGTAAGTTAATTCAATCCCCTTATTATTCT
GACTTGTAAGGGGATTCAAACCCCATCAATGGTTTCTAAACTTAAGTTTCTTATTTTAGAGAGAT
AAGTTTTATTATAAAAAAATGTGTGGGTTTTTTGATTAATGAAGAAACCATCTCCAAAATACTTT
ATATAAGAAATTTTGGAGAGAATTTCTAAATATTTAAGAAACCCACATAATTAAATAATATTATT
TTTGTTGTTTTAATGTTAAGAAACTTATATTTAGAAACCACCAATGAAATTCCTCTAAACATTAA
CATTGCATATAGTCATAAAAAAAATATCTATTTTCTTTTTGGTTCTCTTTTTAATATAGCTTTCT
TGGTCAACACTTATTCTTAAATATCTTAGAAATT

Figure S10. Schematic representation of the retrocopy “R_ AT4G21660.1” encoded by the Arabidopsis reference genome.

The figure convention follows Fig. S3A.

ATAATCAATGTTTTAAATTaAAACACTTATCTATCAATTTAATAtTATCAAAATTAAATTTATTT
TAAAAACTATCTCAAAATATCTATTTTGTTATTTATATATGTTTTAAAATTAAAATAATTATTTA
TTAAATTTAAATTAATTTAAATAATAAAATTAGTATTCACTTTAAATTTTCAATATAATATTTAT
TATCTTATAACATAATAAATGTTTTATAGTTAACGATATATGCATTCAATTAATTTTTAAAAAAT
ATTAAAATATTCTTTTTGTTTTTAAGACTCAAAAGAGTCCCAATTAGGGTTTCTATTTTGCAGAA
CAGAAACAATGGTTTGCGAGAAG**TG**CGAGAAGAAGTTATCGAAGGTGATAGTAGCAGATAAGCAA
TACCACCGAAGGCGGTGGTCGTAAGATGAACGAGAACAAACTCCTCTCTAAGAAGAAAA**GA**TGGA
CTCCTTATGGAAATACAAAGTGCATGATTGCAAGCAGCAAGTACACCAAGATGTCAAGTACTGCC
ACACCTGTGCTTATACCAAA**GG**GGTTTGTGCAATGTGTGGTAAGCAAGTACTTGATACAAAGCTT
TGCAAGCAAAGCAATGTATAATTCAAAGCAGATGCTATTTGGCATGTTGAAGTAGACCTAATGGT
TTAGGGACTCTCGACTGTTTCAAGCTCAGTTGATCACTATCATTAACTTGCAGTATTGAAACTGG
GGTTGTAAAGTTCACCAACTGTTATGTAATGTAGTGGCATGCTTTGTAAGTTTAATTCTTCCTGG
GTAATGAAATATGATGGTAATAACTTCTCTGCATTCTCTCTTTTAACAAAAAAAAAAAAAAAAATT
AAAATATTATCATTTAAAATTAAGTTGATTGTTAAAATTATAATCTATCACTATAATAATTTTTT
ATAATTTTGAGTTAGTTTTTTATTATTTTTCAGAATTTATCAATATTTAAATGTGTTATTTTGAT
AATTTTATATCAAAATTTTGATTTTTAATATATACATATATATATCTCATAATTTACATACTATT
TAATTAATTTAAATAAATTGAAATAATAAAAATTATTATAACACGC

Figure S11. Schematic representation of the retrocopy “R_cassava4.1_019865m” encoded by the *M. esculenta* reference genome.

The figure convention follows Fig. S3A.

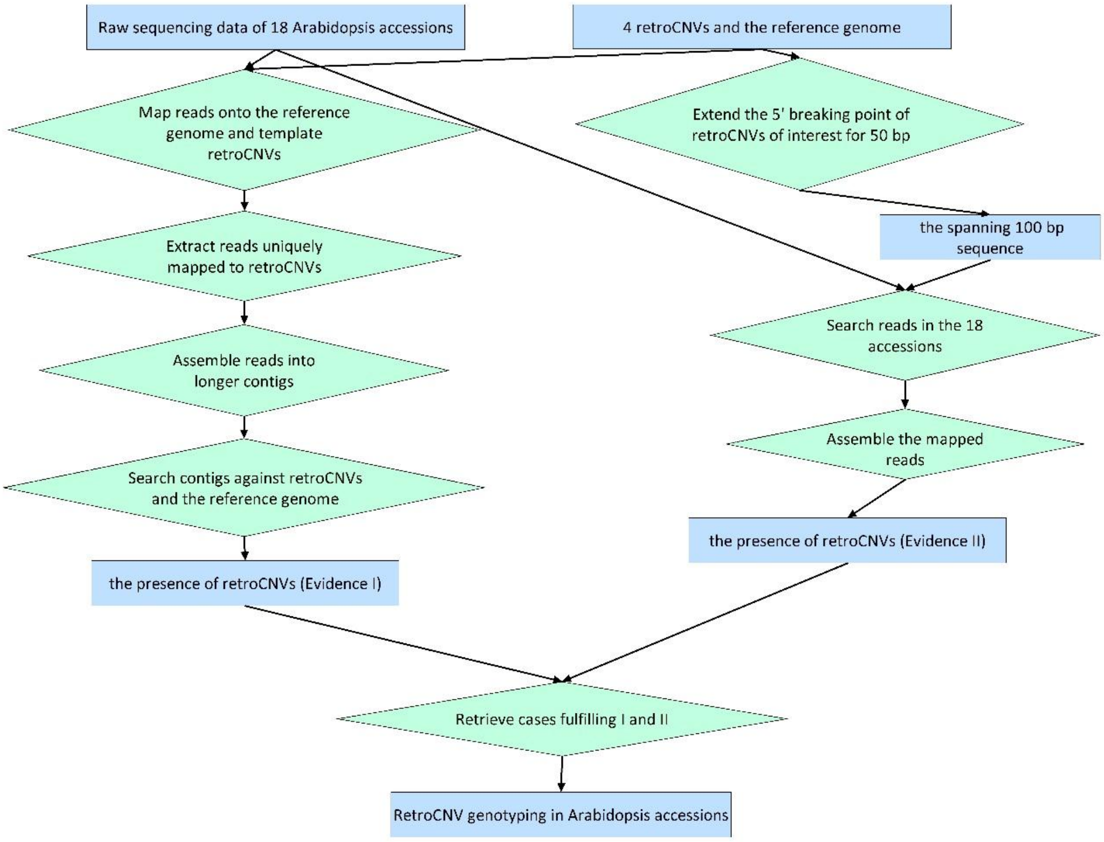

Figure S12. RetroCNV genotyping across Arabidopsis accessions.

References in the Supplementary Information

1. Schrider, D. R., Stevens, K., Cardeno, C. M., Langley, C. H., & Hahn, M. W. Genome-wide analysis of retrogene polymorphisms in Drosophila melanogaster. *Genome Res* **21**, 2087-2095 (2011).
2. Schrider, D. R. *et al.* Gene copy-number polymorphism caused by retrotransposition in humans. *PLoS Genet* **9**, e1003242 (2013).
3. Gan, X. *et al.* Multiple reference genomes and transcriptomes for Arabidopsis thaliana. *Nature* **477**, 419-423 (2011).
4. Huala, E. *et al.* The Arabidopsis Information Resource (TAIR): a comprehensive database and web-based information retrieval, analysis, and visualization system for a model plant. *Nucleic Acids Res* **29**, 102-105 (2001).
5. Poole, R. L. The TAIR database. *Methods Mol Biol* **406**, 179-212 (2007).
6. Chevreux, B. *et al.* Using the miraEST assembler for reliable and automated mRNA transcript assembly and SNP detection in sequenced ESTs. *Genome Res* **14**, 1147-1159 (2004).
7. Kent, W. J. BLAT--the BLAST-like alignment tool. *Genome Res* **12**, 656-664 (2002).
8. Ruby, J. G., Bellare, P., & Derisi, J. L. PRICE: software for the targeted assembly of components of (Meta) genomic sequence data. *G3 (Bethesda)* **3**, 865-880 (2013).
9. Jurka, J. Repbase update: a database and an electronic journal of repetitive elements. *Trends Genet* **16**, 418-420 (2000).
10. Jurka, J. *et al.* Repbase Update, a database of eukaryotic repetitive elements. *Cytogenet Genome Res* **110**, 462-467 (2005).
11. Ouyang, S. & Buell, C. R. The TIGR Plant Repeat Databases: a collective resource for the identification of repetitive sequences in plants. *Nucleic Acids Res* **32**, D360-363 (2004).
12. Prochnik, S. *et al.* The Cassava Genome: Current Progress, Future Directions. *Trop Plant Biol* **5**, 88-94 (2012).
13. Rho, M., Choi, J. H., Kim, S., Lynch, M., & Tang, H. De novo identification of LTR retrotransposons in eukaryotic genomes. *BMC Genomics* **8**, 90 (2007).
14. Rho, M. & Tang, H. MGEScan-non-LTR: computational identification and classification of autonomous non-LTR retrotransposons in eukaryotic genomes. *Nucleic Acids Res* **37**, e143 (2009).
15. Li, W. & Godzik, A. Cd-hit: a fast program for clustering and comparing large sets of protein or nucleotide sequences. *Bioinformatics* **22**, 1658-1659 (2006).
16. Ma, J., Devos, K. M., & Bennetzen, J. L. Analyses of LTR-retrotransposon structures reveal recent and rapid genomic DNA loss in rice. *Genome Res* **14**, 860-869 (2004).
17. Abdelsamad, A. & Pecinka, A. Pollen-specific activation of Arabidopsis retrogenes is associated with global transcriptional reprogramming. *Plant Cell* **26**, 3299-3313 (2014).
18. Zhu, Z., Zhang, Y., & Long, M. Extensive structural renovation of retrogenes in the evolution of the Populus genome. *Plant Physiol* **151**, 1943-1951 (2009).
19. Zhang, Y., Wu, Y., Liu, Y., & Han, B. Computational identification of 69 retroposons in Arabidopsis. *Plant Physiol* **138**, 935-948 (2005).
20. Yang, Z. PAML: a program package for phylogenetic analysis by maximum likelihood. *Comput Appl Biosci* **13**, 555-556 (1997).

21. Betran, E., Thornton, K., & Long, M. Retroposed new genes out of the X in Drosophila. *Genome Res* **12**, 1854-1859 (2002).
22. Robinson, J. T. *et al.* Integrative genomics viewer. *Nat Biotechnol* **29**, 24-26.
23. Thorvaldsdottir, H., Robinson, J. T., & Mesirov, J. P. Integrative Genomics Viewer (IGV): high-performance genomics data visualization and exploration. *Brief Bioinform* **14**, 178-192.
24. Li, H. & Durbin, R. Fast and accurate short read alignment with Burrows-Wheeler transform. *Bioinformatics* **25**, 1754-1760 (2009).
25. Tamura, K. *et al.* MEGA5: molecular evolutionary genetics analysis using maximum likelihood, evolutionary distance, and maximum parsimony methods. *Mol Biol Evol* **28**, 2731-2739.
26. Kumar, S., Tamura, K., & Nei, M. MEGA: Molecular Evolutionary Genetics Analysis software for microcomputers. *Comput Appl Biosci* **10**, 189-191 (1994).
27. Edgar, R. C. MUSCLE: multiple sequence alignment with high accuracy and high throughput. *Nucleic Acids Res* **32**, 1792-1797 (2004).
